# Supplementary material for: The effect of exchanging drawings with peers on the happiness of children with cancer, aged 7–11 years: A clinical trial
Source: PLoS One. 2021 Oct 15;16(10):e0257867. doi: 10.1371/journal.pone.0257867 (PMC8519419; doi:10.1371/journal.pone.0257867)
Supplement: S1 Table — (DOCX) [file pone.0257867.s002.docx]

**S1 Table. A comparison of the demographics variable**

| Demographic Variable | | Control  n (%) | Intervention  n (%) | P- Value |
| --- | --- | --- | --- | --- |
| Gender | Female | 17 (51.5) | 15 (45.4) | *P=.622 |
|  | Male | 16 (48.5) | 18 (54.6) |  |
| Interested in drawing | Yes | 31 (93.9) | 33 (100) | **P= .492 |
|  | No | 2 (6.1) | 0 (0) |  |
| Have a habit of drawing | Yes | 23 (69.6) | 24 (72.7) | *P=.786 |
|  | No | 10 (30.4) | 9 (27.3) |  |
| Duration of illness | Less than a year | 20 (60.6) | 17 (51.5) | P=.457 |
|  | Over a year | 13 (39.4) | 16 (48.5) |  |

*Chi-Square Test **Fisher Exact Test
