# Supplementary material for: The effect of exchanging drawings with peers on the happiness of children with cancer, aged 7–11 years: A clinical trial
Source: PLoS One. 2021 Oct 15;16(10):e0257867. doi: 10.1371/journal.pone.0257867 (PMC8519419; doi:10.1371/journal.pone.0257867)
Supplement: S2 Table — (DOCX) [file pone.0257867.s003.docx]

**S2 Table. A comparison of the happiness scores**

| Happiness | Intervention  Mean ± SD | Control  Mean ± SD | *Independent sample t-test | Effect Size  (95% Confidence Interval) |
| --- | --- | --- | --- | --- |
| Before | 3.15 ± 0.34 | 3.02 ± 0.3 | t=1.62 df=64  p=0.11 |  |
| After | 3.22± 0.22 | 3.11± 0.25 | t=1.932 df=64  p=0.058 | 0.46(0.022,0.956) |
| Mean difference | 0.07 ± 0.23 | 0.08 ± 0.31 | t=0.236 df=64  p=0.814 |  |
| **Paired t-test | t=1.813 df=32  p=.079 | t=1.639 df=32 p=.111 |  |  |

* Independent t-test **Paired T-Test (2-tailed)

Cohen’s Standard Effect Sizes: 0.2 = small; 0.5 = moderate; 0.8 = large
