# Supplementary material for: The effect of exchanging drawings with peers on the happiness of children with cancer, aged 7–11 years: A clinical trial
Source: PLoS One. 2021 Oct 15;16(10):e0257867. doi: 10.1371/journal.pone.0257867 (PMC8519419; doi:10.1371/journal.pone.0257867)
Supplement: S1 File — (DOC) [file pone.0257867.s004.doc]

# بنام خداوند بخشنده مهربان

###### جمهوري اسلامي ايران

### وزارت بهداشت، درمان و آموزش پزشكي

**دانشگاه علوم پزشكي وخدمات بهداشتي درماني تهران**

معاونت پژوهشي – مديريت امورپژوهش

فرم پيش نويس طرح پژوهشي

(PROPOSAL)

عنوان طرح: بررسی تاثیر نقاشی همتایان بر شادکامی کودکان مبتلا به سرطان در سنین مدرسه

نام و نام خانوادگي طرح دهندگان^1^ :

خدیجه زارعی-اکرم السادات حسینی

سمیه پلوان

دانشكده/ مركز تحقيقاتي :پرستاری و مامایی دانشگاه علوم پزشکی تهران

#

قسمت اول- توضيح نكات لازم و ضروري

توجه : پاسخ بسياري از پرسشهاي شما درباره مندرجات اين فرم در مطالب زير ارائه شده است، بنابراين خواهشمند است قبل از تكميل اين فرم مطالب زير رابه دقت مطالعه فرموده ودر نظر داشته باشيد.

1. مطابق با آيين نامه اجرايي طرحهاي تحقيقاتي روند رسيدگي به طرحهاي پيشنهادي در **دانشكدهها و مراكز تحقيقاتي مصوب** دانشگاه و شوراي گسترش دانشگاه هاي علوم پزشكي مشابه يكديگر بوده و در مورد مراكزي كه وابسته به دانشكده ها ميباشند از طريق دانشكده مربوطه عمل ميشود.
2. **خلاصه روند بررسي طرحهاي** تحقيقاتي بدين ترتيب است ، براين اساس اين فرم بايد پس از تكميل جهت بررسي و طي مراحل تصويب به معاونت پژوهشي دانشكده مورد نظر طرح دهنده تحويل شود.

# تكميل و ارائه پيش نويس طرح به معاونت پژوهشي دانشكده و ثبت آن

1. ارائه طرح پيشنهادي به شوراي پژوهشي گروه مربوطه جهت بررسي و و انجام تغييرات احتمالي در گروه مربوطه
2. تصويب موضوع در شوراي پژوهشي گروه
3. ارائه طرح مصوب به شوراي پژوهشي دانشكده جهت ادامه روند بررسي
4. تصويب موضوع در شوراي پژوهشي دانشگده
5. ارائه طرح مصوب دانشكده به حوزه معاونت پژوهشي دانشگاه
6. ثبت در فهرست نوبت شورا و ارجاع به كارشناسان جهت بررسي هاي كلي
7. تكميل اطلاعات و قرارگرفتن طرح در دستور كار شوراي پژوهشي دانشگاه
8. تصويب موضوع در شوراي پژوهشي دانشگاه
9. اعلام به مجري و مقدمات قرارداد …
10. در مواردي كه اجراي طرح پيشنهادي مستلزم **همكاري بخشها يا سازمانهاي ديگر** باشد, طرح دهنده بايد قبلا" نظرموافق سازمانهاي مربوطه را كسب نموده و موافقت نامه كتبي ايشان را ضميمه اين پيش نويس نمايند.
11. كليه طرحهايي كه به تصويب شوراي پژوهشي دانشگاه ميرسد بر اساس قراردادي كه بين معاونت پژوهشي دانشگاه و مجري طرح منعقد مي‏شود قابل اجرا خواهد بود. بنابراين **معاونت پژوهشي دانشگاه هيچگونه مسئوليتي در برابر فعاليتهاي قبل ازتصويب طرح وآنچه كه خارج ازمحدوده قرارداد منعقده انجام پذيرد نخواهد داشت**.
12. طرح دهندگان ملزم به رعايت كليه **ضوابط و قوانين مندرج در آئين نامه طرحهاي تحقيقاتي دانشگاه** علوم پزشكي تهران ميباشند. لذا پيشنهاد ميگردد تا مجريان و طرح دهندگان محترم جهت آگاهي ازمفاد آيين نامه مذكور به معاونين پژوهشي دانشكده ها يا مراكز تحقيقاتي مراجعه نمايند.
13. چنانچه انجام طرح پژوهشي در مرحله‏اي از پيشرفت آن اعم از اينكه به نتيجه نهايي رسيده يا نرسيده باشد، **منجر به كشف يا اختراع و يا تحصيل حقوقي شود**، مجري طرف قرارداد موظف است مراتب را كتبا" به معاونت پژوهشي دانشكده ذيربط ويا معاونت پژوهشي دانشگاه اطلاع دهد. در اين رابطه حقوقي كه در اثر اجراي طرح تحقيقاتي ايجاد گرديده است با توجه به متن قرارداد منعقده و يا متمم آن مشخص ميگردد.
14. در صورت تمايل مجري به انتشار يا ارائه نتايج حاصله در داخل يا خارج از كشور ( بجز نشريات دانشگاه ), لازمست قبلا" نظر موافق معاونت پژوهشي دانشگاه را در اين زمينه جلب نمايد. بديهي است كه **ذكر حمايت مالي و همكاري دانشگاه در اجراي طرح براي انتشارات** مذكور الزامي خواهد بود.
15. كليه **تجهيزات و لوازم مصرفي و غيرمصرفي باقيمانده** از اجراي طرح كه از محل اعتبار آن تهيه شده است، پس از اتمام اجراي طرح متعلق به دانشگاه بوده وهرگونه تصرفي در آنها منوط به كسب مجوزهاي قانوني است.
16. در صورتيكه قراردادي در مورد تجهيزات و لوازم و موادي كه از محل اعتبار پژوهشي تهيه شده است بين پژوهشگر و سازمانهاي ديگر و دانشگاه منعقد شده باشد مطابق اين قرارداد عمل خواهد شد.
17. در صورتيكه هريك از بندهاي 2و3و4 و 12و … رعايت نشود بررسي طرح ممكن نبوده و مسئوليتي از اين بابت متوجه شوراهاي پژوهشي گروه دانشكده و دانشگاه نمي باشد.

**راهنماي تكميل اين فرم**

1. اين فرم بايد به زبان فارسي (ودرصورت لزوم انگليسي) تايپ شده و فاقد هرگونه ابهامي، تكميل گردد. بنابر اين **معاونت پژوهشي دانشكده و يا دانشگاه ميتواند از پذيرفتن فرمهايي كه به نحو نامطلوب تكميل شده است، خودداري نمايند.**
2. كليه قسمتهاي فرم بايد به نحو مناسب تكميل شده و طرح دهنده بايد به كليه نكات وتذكرات متن فرم توجه كامل داشته باشند تا**هيچ موردي بي جواب و بدون علامت و توضيح نباشد**.
3. در پاره اي از موارد كه فضاي كافي براي توضيحات مد نظر طرح دهنده وجود ندارد، وي ميتواند **توضيحات اضافه را در برگه أي بصورت ضميمه و با اشاره** به بند و قسمت مورد نظر به فرم اضافه نمايند. چنانچه طرح دهنده از شكل رايانه اي اين فرم استفاده مينمايد هيچ محدوديتي در فضاهاي پيش بيني شده وجود ندارد.
4. براي ارائه فرم رايانه اي طرح ، اين فرم بايد در قالب نرم افزار Word 95(arabic edition) و يا ويرايش هاي بالاتر از آن باشد.
5. هنگام ارائه اين فرم تكميل صفحه **خلاصه مشخـصات طرح الزامي است.**
6. هنگام ارائه اين فرم **جدول همكاران اصلي طرح** (بند 12) **بايد تكميل شده و به امضا و تاييد فرد مورد اشاره رسيده** باشد.
7. لازم است طرح دهنده **براي تكميل قسمت روش اجراي طرح** به جدول نوع تحقيق ( بند23) توجه كامل داشته و **كليه موارد اشاره شده** در بند مربوطه را توضيح دهد.
8. لازم است طرح دهنده توضيح كاملي در رابطه با ابزار جمع آوري اطلاعات (پرسشنامه و يا …) ارائه نموده و نمونه اي از آن را ضميمه نمايد.
9. در صورتيكه ملا حظات اخلاقي براي اجراي طرح وجود دارد لازم است طرح دهنده توضيح كاملي در باره اين نكات ارائه نموده و نمونه اي از رضايت نامه مورد استفاده براي طرح را ضميمه نمايد.
10. در صورتيكه محدوديتهايي براي اجراي طرح تصور ميشود لازم است طرح دهنده به اين محدوديتها اشاره نموده و توضيح كاملي براي مقابله با اين محدوديتها ارائه نمايد.
11. زمان شروع طرح، بعد از تصويب آن بوسيله شوراي پژوهشي دانشگاه و با هماهنگي مدير اجرايي طرح و حوزه مديريت امور پژوهشي، از هنگام تامين اعتبار در نظر گرفته ميشود
12. دريافت گزارشهاي علمي و اجرايي طرح با توجه به جدول گانت طرح مصوب صورت ميپذيرد. براين اساس لازم است طرح دهنده در جدول مذكور زمان ارائه گزارشهاي پيشرفت طرح و محتواي پيش بيني شده گزارش خود را مشخص نمايد.
13. هزينه هاي كارمندي (پرسنلي) با توجه به **حجم فعاليت** (در نظر گرفتن ساعات كار براي افراد شاغل در طرح) و **جدول زمان بندي** اجراي طرح و **پيوست شماره يك** (كه فعلا مد نظر دانشگاه قرار دارد) تكميل ميشود.
14. طرح دهنده بايد توجه داشته باشد كه حد اكثر ساعات كار براي افراد شاغل در طرح مطابق قوانين اداري واستخدامي 64 ساعت در هر ماه است. رقم حق الزحمه ساعتي افراد با توجه به موقعيت استخدامي و رتبه علمي، در پيوست شماره يك آمده است. بنابر اين طرح دهنده ميتواند با تغيير در ساعات كار رقم كلي حق الزحمه افراد همكار را تغيير دهد
15. **هرگونه نقص يا اشتباهي درمحاسبه هزينه هاي** پيش نويس كه در تصميمات متخذه دخالت داشته باشد به **عهده تكميل كننده فرم است** و دانشگاه تعهدي در خصوص تامين كسري موارد اشتباه شده ندارد.
16. در موارديكه مجري محترم در تكميل اين فرم نيازمند **راهنمايي** باشد ، اين حوزه با هماهنگي قبلي به **نشاني انتهاي اين صفحه** در خصوص راهنمايي‏هاي مورد نظرآمادگي كامل دارد.

### توضيح مفاهيم

| شماره | كلمه | مفهوم |
| --- | --- | --- |
| 1 | طرح دهندگان | فرد يا افرادي هستند كه پيشنويس طرح را تهيه نموده و معمولا اجراي تحقيق نيز بوسيله ايشان انجام ميپذيرد. بر اين اساس و با توجه به آيين نامه طرحهاي تحقيقاتي در اين نوشتار عبارات مجريان يا مجريان اصلي و طرح دهندگان معادل يكديگرهستند. |
| 2 | مدير اجرايي طرح | فردي است كه ازبين مجريان طرح انتخاب شده ومسوليت اجراي طرح از نظر مالي ، حقوقي واداري بعهده ايشان است. بين مدير اجراي طرح وسايرمجريان امتيازخاصي وجود ندارد و مديراجراي طرح صرفا مسئول اجرايي وطرف مذاكره و عامل اجراي طرح شناخته ميشود.. يك طرح تحقيقاتي نميتواند بيش از يك مديراجرايي داشته باشد. |
| 3 | همكاران اصلي طرح | همكاراني هستند كه حضور شخص يا همكاري تخصص ايشان در انجام طرح ضروريست |
| 4 | طرح كاربردي | طرحي است كه داراي نتايج بالفعل بوده و نتايج حاصل از انجام آن بلا فاصله پس از اتمام طرح قابل استفاده باشد |
| 5 | طرح بنيادي | طرحي است كه داراي نتايج بالقوه بوده و نتايج حاصل از انجام آن بلا فاصله پس از اتمام طرح قابل استفاده نباشد |
| 6 | طرح جامعه نگر | طرحي است كه بر اساس نياز بهداشتي درماني جامعه ، در تلاش براي رفع مشكل ويا يافتن پاسخ سوال مشخصي باشد |
| 7 | HSR | Health System Research تحقييقاتي را شامل ميشود كه در قالب طرحهاي جامعه نگر ارائه ميشوند |
| 8 | هدف اصلي طرح | general objective هدفي است كه طرح در انتها بدنبال دستيابي به آن است. اين هدف بايد با انجام طرح قابل حصول باشد |
| 9 | اهداف فرعي طرح | Specific objectives اهدفي هستند كه قبل يا همگام با هدف اصلي طرح حاصل خواهند شد. |
| 10 | اهدف كاربردي طرح | Applied objectives اهدافي هستند كه بصورت عملي پس از انجام طرح بدست آمده و جزو نتايج بالفعل طرح ميباشند. |
| 11 | فرضيات | Hypothesis انتظارات و پيشفرضهايي است كه طرح دهنده بر اساس آن اقدام به تنظيم پيش نويس طرح نموده است. |
| 12 | ملاحظات اخلاقي | Ethical points Or Ethics Considerations كليه اصول و مباني اخلاقي، انساني، مذهبي، و… است كه بايد در حين پژوهش از سوي آزمايشگر بر روي آزمودني اعم از انسان و حيوان و … رعايت شود |
| 13 | جدول زمان بندي مراحل اجراي طرح | Gantt Chart جدولي است كه طي آن محقق ابتدا و انتهاي فعاليتهاي اجرايي و طول زمان مراحل مختلف پژوهش را در آن مشخص ميكند. |

**قسمت دوم - خلاصه مشخـصات طرح**

عنـوان طرح : بررسی تاثیر نقاشی همتایان بر کودکان مبتلا به سرطان در سنین مدرسه

مدير اجرايي طرح^2^ :

دانشـكده / مركز تحقيقاتي : دانشکده پرستاری و مامایی تهران

گـروه :کودکان و مراقبت ویژه نوزادان

محيط پژوهش: بیمارستان مرکز طبی کودکان و مدرسه تربیت

مدت اجرا : 12 ماه

خلاصه ضرورت اجرا :

امروزه، به دلیل پیشرفت­های زیاد در زمینه تشخیص زودرس، تکنولوژی آزمایشگاهی پیشرفته، آسیب شناسی و روش­های درمانی کارا و موفق، کودکان مبتلا به سرطان امکان زندگی طولانی­تری دارند. این درحالی است که بیماری نه فقط به عنوان یک عامل تنش­زا سلامت جسمانی کودک را به مخاطره می­اندازد، بلکه به­عنوان عامل تهدید کننده سلامت روانی تلقی می­شود که در همه زمینه­های روحی، روانی، اجتماعی، فرهنگی و عاطفی زندگی بیمار تداخل ایجاد می­کند. در این رابطه، مداخلات تکمیلی نظیر بکارگیری هنر در کودکان مبتلا به سرطان، می­تواند باعث افزایش توانایی آنان در ارتباط با مسایل همراه با بیماری باشد و بیمار را جهت شرکت فعال در روند درمان، تشویق نماید تا برای کنترل شرایطی که با آن مواجه شده است قابلیت لازم را کسب کند. نقاشی به عنوان یکی از شیوه­های استفاده از هنر در ارتقاء سلامت روان کودکان، مورد توجه قرار گرفته است . تحقیقات نشان داده است با آموزش نقاشی توسط همتایان و تشکیل کلاس می­توان شادی را در کودکان مبتلا به سرطان افزایش داد. در این صورت خلا مدرسه نرفتن درکودکان مبتلا به سرطان را کاهش داده و همچنین موجب افزایش اعتماد به نفس و شادکامی در میان کودکان تحت درمان سرطان خواهد شد. به­طوری که کودک خود را در محیطی کاملا آزاد حس خواهد کرد و در نتیجه در روند درمانی کمک بیشتری برای بهبود کودک انجام خواهد شد. در کودکان سن مدرسه همتایان نقش مهمی در تکامل روانی و ارتقا وضعیت روانشناختی آنان دارندو پتانسیلی در دسترس و موثر است که کودکان را قادر می سازد به یکدیگر یاری رسانند. بنابراین این مطالعه در پی آنست تا تاثیر نقاشی همتایان را بر شادکامی کودکان مبتلا به سرطان در سنین مدرسه ارزیابی کند. در صورتی­که نتایج این تحقیق موثر باشد هنر و بخصوص نقاشی می­­تواند به عنوان یک مداخله پرستاری در جهت ارتقا سلامت کودکان بستری در بیمارستان مورد استفاده قرار گیرد. از طرفی از کودکان همتا به عنوان نیرویی مشتاق و کارآمد در جهت ارتقا وضعیت روانشناختی کودکان می توان استفاده کرد.

**خلاصه روش اجـراي طرح :**

پژوهش حاضر کارآزمایی بالینی تصادفی است که تاثیر متغیر مستقل (مداخله نقاشی همتایان) بر متغیر وابسته (شادکامی) در کودکان مبتلا به سرطان بستری بررسی خواهد شد.به شیوه در دسترس 33 کودک هفت تا 11 ساله مشغول به تحصیل را به­عنوان گروه همتا و66 کودک مبتلا به سرطان (با استفاده از روش تخصیص تصادفی بلوکی 33 کودک مبتلا به سرطان درگروه مداخله و 33 کودک در گروه کنترل قرار می­گیرند) انتخاب کرده با رضایت آگاهانه و بر اساس معیارهای ورود کودک مبتلا به سرطان باید :1 . در دوره سنی 11-7 سال ودارای پرونده در بیمارستان؛ 2. قادر به درک و پاسخگویی به سوالات باشد، 3.کودک معلولیت جسمانی نداشته باشد تا توانایی کشیدن نقاشی را داشته باشد. معیارهای خروج نیز شامل:1. کودکانی که قادر به شرکت در جلسات نقاشی نباشند،2. فوت کودک

معیار ورودهمتایان:1- سن 11-7 و2- مشغول به تحصیل و3- نداشتن معلولیت مانع کشیدن از نقاشی ،می باشد

.مکان پژوهش: بیمارستان کودکان ۱۷ شهریور رشت خواهد بود. برای شروع مداخله، ابتدا از همتایان مدرسه ای با در اختیار قراردادن لوازم مورد نیاز نقاشی از جمله برگه،مداد نقاشی،مداد شمعی،پاک کن و... خواسته می شود تا برای همتایان بیمارشان با موضوع آزاد و زمان نامحدود نقاشی بکشند.این فرآیند برای 5 جلسه و با فاصله یک هفته بین جلسات انجام میشود،سپس نقاشی ها توسط محقق به گروه مداخله کودکان بیمار داده میشود و از آنها درخواست میگردد تا برای همتایان سالمشان با هر موضوعی که خواستند نقاشی بکشند. 5 جلسه برای هر یک از گروه­ها (گروه همتایان 5 جلسه و 5 جلسه نیز برای گروه کنترل و گروه مداخله) و با فاصله یک هفته. شرایط نقاشی و وسایل در اختیار کودکان بیمار مشابه کودکان همتا در مدرسه است. برای گروه کنترل نیز اقدامات روتین بخش انجام خواهد شد. پرسشنامه در کودکان بیمار در هر دو گروه (مداخله و کنترل)،یکبار در ابتدای مطالعه و بار دوم در انتهای هفته پنجم تکمیل خواهد شد. بر اساس پرسشنامه شادکامی کودکان روند تغییرات میان آن­ها قبل و بعد از مداخله برای هر دو گروه مورد بررسی قرار خواهد گرفت .این پرسشنامه توسط پژوهشگر مورد بررسی قرار خواهد گرفت؛ سپس با استفاده از نرم افزار SPSS روابط میان آن­ها و همچنین متغییر­ها با تست­های آماری همچون تحلیل کوواریانس و آزمون t مورد بررسی قرار می­گیرد.

خلاصه هزينه ها

| هزينه پرسنلي | 10000000ريال | هزينه مسافرت | 0 ريال |
| --- | --- | --- | --- |
| هزينه آزمايشات و خدمات تخصصي | 0 ريال | هزينه هاي ديگر | 2000000 ريال |
| هزينه مواد و وسايل مصرفي | 5000000 ريال | جمع كل | ........... ريال |
| هزينه وسايل غير مصرفي | 0 ريال |  | 17000000 ريال |

# قسمت سوم- اطلاعات مربوط به عوامل اجرايي طرح

**توجه : چنانچه طرح دهنده بيش از يكنفر باشد لازم است هركدام از ايشان بطورجداگانه اين قسمت را تكميل نمايند.**

1. نام و نام خانوادگي طرح دهنده :**اکرم السادات سادات حسینی**
2. رتبه علمي:
3. محل خدمت: دانشکده پرستاری مامایی دانشگاه علوم پزشکی تهران
4. نشاني محل خدمت: تهران – ميدان توحيد – دانشكده پرستاري و مامايي دانشگاه علوم پزشكي تهران
5. تلفن محل خدمت:
6. نشاني پست الكترونيك:
7. نشاني يا تلفن براي دسترسي سريع و پيامهاي فوري:
8. درصورتيكه طرح دهنده داراي سمتهاي اجرايي در داخل يا خارج محيط دانشگاه ميباشند جدول زير را تكميل نمايند

| **عنوان سمت** | **نشاني محل كار** | **تاريخ شروع فعاليت در اين سمت** | **تلفن محل كار** |
| --- | --- | --- | --- |
|  |  |  |  |
|  |  |  |  |
|  |  |  |  |

1. **درجات علمي و سوابق تحصيلي طرح دهنده به ترتيب از ليسانس به بعد ذكر گردد**

| **درجه تحصيلي** | **رشته تحصيلي وتخصصي** | **دانشگاه يا محل تحصيل** | **كشور** | **سال دريافت** |
| --- | --- | --- | --- | --- |
|  |  |  | ايران |  |
|  |  |  | ايران |  |
|  |  |  | ايران |  |

1. آيا تا كنون دوره هاي روش تحقيق را گذرانده ايد؟ بلي -- خير-- در صورت پاسخ مثبت، جدول زير را تكميل فرماييد.

| **سطح دوره ونوع آموزشهاي ارائه شده** | **تاريخ** | **محل برگزاري دوره** |
| --- | --- | --- |
|  |  |  |

سابقه طرحهاي تحقيقاتي كه طرح دهنده قبلا در آن همكاري داشته ويا هم اكنون درحال اجرادارد ، ذكر شود.

| **عنوان طرح** | **محل اجرا** | **مدت اجرا** | **وضعيت طرح** | **نوع همكاري** |
| --- | --- | --- | --- | --- |
|  |  |  |  |  |
|  |  |  |  |  |

# مشخصات همكاران اصلي^3^ طرح :

| رديف | نام و نام خانوادگي | شغل | درجه علمي | نوع همكاري | امضاي همكار |
| --- | --- | --- | --- | --- | --- |
| 1- |  |  |  |  |  |
| 2- |  |  |  |  |  |

**قسمت چهارم – اطلاعات مربوط به طرح پژوهشي**

1. عنوان طرح به فارسي :
2. بررسی تاثیر نقاشی همتایان بر شادکامی کودکان مبتلا به سرطان در سنین مدرسه

عنوان طرح به انگليسي:the effect of painting by peers on happiness in school-aged children with cancer

1. نوع طرح :

كاربردي^4^ بنيادي بنيادي-كاربردي جامعه نگر^6^(HSR) ^7^

1. بيان مسئله و ضرورت اجراي طرح : (درصورت نياز مي­توانيد از صفحات اضافه استفاده نماييد.)

علی­رغم پیشرفت در شناسایی و درمان بدخیمی­ها، سرطان دومین عامل مرگ و میر در کودکان است . .طی سال­های مختلف در کشور ما شمار کودکان مبتلا به سرطان از نه کودک در هر 100 هزار کودک در سال به 15 کودک در سال 2008 افزایش یافته است . با توجه به اینکه هرگونه تغییر و بیماری در کودک، سلامتی او را به مخاطره می اندازد، سبب عدم ارضای نیاز­های کودک خواهد شد .ابتلا به سرطان تأثیرات روان­شناختی عمیقی بر کودکان بجا می­گذارد. از طرفی بستري شدن در بیمارستان و حضور مداوم در محیط سرد و بی­روح بیمارستان، کودك را غمگین و تنیده می­کند همچنین درمان­هاي طولانی مدت و دردآور این بیماري، شدت مشکلات روانی موجود را افزونتر می­کند. بستری شدن برای کودکان یک بحران در سازگاری با شرایط محیط جدید است آن هم در شرایطی که نه تنها از سلامت برخوردار نیستند بلکه در معرض برخورد با عوامل ناشناخته و خطر آفرین نیز قرار دارند. بنابراین بستری شدن کودک در بیمارستان سبب اضطراب ناشی از جدایی، اندوه، ترس از محیط جدید و ترس از ناتوانی می­شود.مطالعات متعدد نشان داده­اند که افراد با سن پایین­تر مبتلا به سرطان تحت شیمی درمانی در معرض خطر مشکلات روانی در دو حیطه سازگاری اجتماعی با همسالان و احساس خوب بودن، هستند غالباً بیماری و بستری شدن در بیمارستان­ها اولین بحرانی است که کودکان با آن مواجه می­شوند .گزارش­های آماری نشان داده است که 50-80 درصد از بیماران مبتلا به سرطان به طور همزمان از مشکلات روانی رنج می­برند. نشان داده شده است که کودکان نجات یافته از سرطان دارای مشکلات روانشناختی بوده­اند ­بطوریکه براساس شاخص جهانی[[1]](#footnote-2) حساسیت در روابط متقابل، افسردگی و خشونت در آن­ها افزایش یافته است . بنابراین لازم است تا شیوه هایی بکار گرفته شود که میزان این عوارض و مشکلات را به حدافل برساند. از آنجا که كودكان با ايجاد ارتباط با ديگران خود را مى­شناسند، نظرات و عقايد آن­ها درباره خودشان بسيار تحت تأثير نظرات اطرافيان آن­هاست.کودکان معمولا به­واسطه نظر ديگران است كه مى­فهمند، فرد خوبى هستند يا نه، آيا دوست داشتنى هستند يا نه. كودكانى كه نسبت به خود احساس خوبى دارند، خودپنداره مثبتى را در درون خود پرورش داده­اند؛ و نسبت به ديگر كودكان ارتباط بهترى با بقيه برقرار مى­كنند، آدم­هاى شادى هستند و به موفقيت مى­انديشند(14). احساس خودپنداره كودكان زمانى رشد پيدا مى­كند كه در مى­يابند افراد نزديكى كه با آن­ها ارتباط دارند، به آن­ها توجه مى­كنند، درباره شان فكر مى­كنند و از بودن با آن­ها لذت مى­برند و مى­خواهند كه در كنارشان باشند. (14)از آنجا که جهت حل مشکلات کودکان باید با آن­ها به زبان خودشان و به شیوه خودشان برخورد شود تا تاثیر دلخواه گرفته شود و هنر یکی از شیوه های گفتاری در کودکان است.(15)کاربرد هنر به­عنوان یک شیوه درمانی در اختلالات روانشناختی، نظر محققان را به خود جلب کرده است؛ بسیاری از پژوهشگران، روانشناسان و متخصصین رشته­های مربوطه از گذشته­های دور تا­کنون در صدد آن بوده­اند که در کنار درمان­های متداول جسمی و روانی، از هنر نیز در قالب ابزاری جهت درمان روحی و درونی که منتج به بهبود جسم می­گردد، بهره مند شوند. هنر به­عنوان پدیده­ زیباشناختی از قدرت خاصی برخوردار است به گونه­ای که می­تواند نیروهای متعارض درون فردی و بین فردی را بهبود بخشیده و سازگاری بهتری برای صاحب اثر ایجاد نماید. به وسیله هنر می توان درک و فهم حالات هیجانی و عاطفی بیمار را تسهیل کرد.

استفاده از هنر خود شامل طیف گسترده­ای از کاربرد­های عناصر هنر نظیر تئاتر، نقاشی، موسیقی و رنگ می­باشد که در یک سوی این طیف، هنر به­عنوان وسیله­ای برای ارتباط غیر کلامی موثر است و در سوی دیگر وسیله­ای در جهت ارتقا وضعیت روانشناختی کودکان می­باشد.(18،19) در رابطه باقدرت هنر، مطالعات گوناگونی انجام گرفته است و بررسی­ها نشان می­دهد که یکی از هنرهای مهم مورد استفاده در کودکان، که می­توان بسیار مفید باشد هنر­های تجسمی است. یکی از شاخه­های هنرهای تجسمی نقاشی است(17،20).)10) به عبارت دیگر، نقاشی فرصتی برای کودکانی که توانایی بیان افکار خود در قالب کلمات به طور طبیعی ندارند را فراهم می­کند . نقاشی ابزاری است برای برون­فکنی احساسات کودکان؛ بدین معنا که کودکان بینش و دنیای درون خود را از طریق تصاویر برون­فکنی می­کنند. نقاشی کودکان، ناب­ترین جلوه طبیعت زیبای کودکانه آن­هاست و از این رو همچون خود آنان با معنی و پر ارزش است. آنان با هر اثر خود، بخشی از هستی خویش را به نمایش می­گذارند.(21) با توجه به موارد گفته شده مشخص است که میتوان از هنر برای ارتقا وضعیت روانشناختی کودکان استفاده کرد. یکی از مشکلات کودکان بستری اندوه ناشی از بستری و دوری از دوستان و مدرسه است. بنابراین ارتقا وضعیت روانشناختی کودکان در این بعد مشکلی است که تا بحال از آن غفلت شده است. یکی از راههای ارتقا وضعیت روانشناختی کودکان توجه به شادی و شادکامی کودکان بستری است. باربارا فردريكسون[[2]](#footnote-3) اظهار مى­دارد كه هيجانات مثبت در تكامل انسان نقش به سزايى دارند. افكار هيجانى مثبت محدوده­ى گسترده­اى از افكار، حركات، بازى، اكتشاف و خلاقيت را در فرد به­وجود مى­آورد. (22)این موضوع بیان کننده نقش شادی در رشد و تکامل کودک می­باشد در نتیجه با استفاده از هنر می­توان این رشد و تکامل را برای کودکان بستری در بیمارستان­ها نیز به ارمغان آورد. در مطالعه مالچيودى[[3]](#footnote-4) (2003) از هنر­هاى خلاقى مثل نقاشى و مجسمه سازى با خاك استفاده گردید که با توجه به مشاهدات وى متوجه شد خلاقيت هنرى موجب اميدوارى، عزت نفس، استقلال، حس رقابت و بيان احساسات در كودكان مبتلا به بيمارى جسمى مى­شود. ايشان عنوان مى­كنند در فرايند استفاده از هنر در كودكانى كه مبتلا به بيمارى هستند کودکان مى­توانند ادراك­ها، نياز­ها و آرزوهاى خود را انتقال دهند.در نهایت ايشان اشاره مى­كنند که با استفاده از هنر مى­توان كودكان را به محيط بيمارستان خو داد و با به نمايش گذاشتن كارهاى هنرى آن­ها، حس اعتماد به نفس، پذيرش، امنيت و دوستى را در آن­ها ايجاد كرد. همچنین می­توان حس سرزندگی، خوش بینی و امید را در میان این کودکان تقویت کرد. سرزندگی و هیجانی که به عنوان احساس اشتیاق، طراوت و سرشار بودن از انرژي شناخته شده، عامل روانشناختی مهمی است که باعث ارتقاي سلامت بیماران مزمن شده و خطر مشکلات روانی ناشی از بیماري را کاهش می­دهد در حقیقت استفاده از هنر با رفع مشکل، حل مسئله و برون ریزی احساسات به پایان می­رسد و این پایان خوش همیشه با شادکامی، نشاط و احساس شعف کودکان همراه است.(25)

یک از انواع آموزش به بیماران، آموزش توسط همتایان می­باشد که در تحصیل و پیشرفت بهداشت و ایجاد محیطی برای یادگیری تاثیر بسزایی دارد. آموزش همتا؛ تبادل اطلاعات، نگرش و رفتار به­ وسیله کسانی است که به­طور تخصصی در آن مورد تربیت نشده باشند . موثر بودن رویکرد آموزشی گروه همسالان بر این تئوری استوار است که اطلاعات حساس، راحت­تر بین افراد هم سن و سال مطرح می­شود. از مزایای رویکرد گروه همسالان می­توان به مواردی نظیر بهبود هنجارهای اجتماعی، ارزش­های حمایت کننده نگرش­های مثبت و رفتارهای بهداشتی، شناخت خوب همسالان از محیط اجتماعی و فرهنگی گروه هدف، درگیر شدن در طراحی طرح­های مربوط به خودشان اشاره کرد . همچنین همسالان با ارتقای سطح همدلی و اعتماد، رابط قویی بین سیستم بهداشت و سایر همسالان می­باشند . با توجه به اهمیت ارتباط با همتایان در دوران کودکی، حمایت همتا به عنوان یک هدف بالقوه در مداخلات درمانی و یا مراقبتی می­تواند در نظر گرفته شود . دوره کودکی دوره تأثيرپذيري از افراد همسال و تقليد از آن­ها است و همسال به عنوان يك مدل در دسترس، روي افراد همسن خود اثر دارد. .[[4]](#endnote-2)در این مطالعه[[5]](#endnote-3)با برگزاری کلاس­های نقاشی توسط همتایان شاید بتوان خلاء مدرسه نرفتن را در آن­ها پر کرد به­طوری که این احساس در آن­ها تقویت شود که هنوزهم در کلاس هستند و تجربه سر کلاس بودن و ارتباط با همسالانشان را داشته باشند. از طرف دیگر پرستار با آشنایی به خصوصیات شخصیتی کودک می­تواند در روند بهبود کودک تحت درمان کمک شایانی بکند.. بنابراین همتا به عنوان عنصری در دسترس و مشتاق کمک می تواند در ارتقا وضعیت روانشناختی کودکان به شدت موثر باشد. با توجه به همه موارد گفته شده می توان نتیجه گرفت استفاده از همتایان در قالب نقاشی به عنوان شیوه ای از بکارگیری هنر در ارتقا سلامت کودکان ممکن است سبب ارتقا وضعیت روانشناختی و شادکامی کودکان مبتلا به سرطان گردد بنابراین این مطالعه در پی آن است که تاثیر کشیدن نقاشی توسط همتایان بر شادکامی کودکان مبتلا به سرطان را بررسی نماید.

چارچوب پژوهش:

چارچوب این پژوهش پنداشتی بوده و بر مفاهیم شادکامی و نقاشی استوار می­باشد. که بر اساس مفهوم شادکامی زیرمفاهیمی چون تعریف شادکامی، شادکامی در کودکان و بر اساس مفهوم نقاشی زیر مفاهیمی مانند تعریف نقاشی، فواید نقاشی، تاثیر آن بر افزایش شادکامی در کودکان مطرح می­شود. که در این راستا نقاشی موجب بهبود زودتر این کودکان و افزایش شادکامی در آن­ها می­شود. بطور کلی در این پژوهش در مورد تاثیر نقاشی توسط همتایان و تاثیر آن بر افزایش شادکامی کودکان بحث خواهد شد.

**سابقه طرح و بررسي متون :**

**تحقیقات صورت گرفته در ایران**

برای بررسی متون در این مطالعه بانک­های اطلاعاتی مختلفی شامل: Pubmed، Scopus، Web of Science، و Scholar مورد بررسی قرار گرفتند. جستجوی کلمات کلیدی ‘Happiness’، ‘painting’، ‘peers’، ‘school-aged children’ و ‘cancer’ ابتداً در عنوان، چکیده و کلید واژه­های این بانک­های اطلاعاتی به صورت انگلیسی و بدون در نظر گرفتن بازه زمانی انجام گرفت. همچنین جستجوی کلمات کلیدی (شادکامی، نقاشی، همتایان، کودکان سن مدرسه و سرطان) نیز در پایگاهای اطلاعاتی داخلی شامل Iranmedex، Magiran، SID، Scholar و Google به صورت فارسی و بدون در نظر گرفتن بازه زمانی صورت پذیرفت.

تا کنون، هنردرمانی را براي بیماری­های مختلفی نظیر بیماران مزمن ، براي کودکان مبتلا به دیابت و براي کودکان بستري در مرکز طبی **کو**دکان تهران انجام گرفته است و نتایج معناداری مشاهده شده است.

**تحقیقات صورت گرفته در ایران:**

امروزه مقایسه مراقبت­ها به­خصوص الگوی مراقبت همتا­محور مورد توجه قرار گرفته است. مطالعه­ای مروری توسط کاظمی و همکاران )1392( با هدف بررسی تعیین اثربخشی مداخلات همتا­محور در مدیریت بیماری دیابت نوع یک در کودکان و نوجوانان با استفاده از پایگاه­های اطلاعاتی مختلف انجام شده است. معیارهای ورود مطالعات انجام شده در این مرور نظام­مند شامل مقالات همتامحور در دیابت نوع یک در کودکان و نوجوانان زیر بیست سال بوده است. نتایج نشان داده است که مداخلات همتا محور می­تواند دارای تاثیر مثبت باشد . بر اساس نتایج این مطالعه، مشارکت همتایان و دوستان می­تواند در آموزش به بیمار به عنوان یک روش موثر استفاده گردد. به علاوه، آموزش و مشارکت همتایان دامنه و ابعاد گسترده­ای دارد که پرستاران و آموزگاران می­توانند از آن برای تامین و ارتقای سلامت کودکان و نوجوانان دارای دیابت و همچنین سایر کودکان بستری همچون کودکان مبتلا به سرطان استفاده نمایند. از تاثیر مثبت تعامل و مشارکت همتایان در این تحقیق همتا محور جهت پیشبرد اهداف پژوهش حاضر استفاده می شود.بنابراین از نیروی بسیار موثر همتایان درایجاد و افزایش شادکامی کودکان استفاده می شود.

نادری و همکاران (1388) تأثير هنر درمانى بر خودپنداره تأييدجويى و شادكامى كودكان مراجعه كننده به مراكز مشاوره و درمان شهرستان اهواز را مورد بررسی قرار داده­اند. نتایج حاصل از این مطالعه نشان داده است که مداخله هنر درمانى در افزايش تأييدجويى، خودپنداره و شادكامى كودكان گروه آزمايش نسبت به گروه گواه تأثير داشته است. آن­ها بیان داشته­اند که خلق اثر هنرى و لذت بردن از آن هر دو مى­تواند علاوه بر رشد باعث فرح و شادى كودك گردد. همچنین، آزادانه بودن فضاى هنرى نيز به شادى و نشاط كودكان افزوده است . بر اساس نتایج و یافته­های حاصل از این مطالعه، پیشنهاد شده است که مطالعه­ در فضای آزد هنری برای کودکان اجرا گردد تا بیشترین تاثیر را داشته باشد. پیش­بینی می­شود بتوان این آزادانه بودن فضای هنری را در مطالعه حاضر با استفاده از حضور همتایان بیشتر مشاهده نمود.بر اساس یافته های این مطالعه ،از ایجاد فضای هنری (شرکت کودکان مبتلا به سرطان در فعالیت های نقاشی در محیط بخش های درمانی بیمارستان) جهت ایجاد و ارتقا اعتماد به نفس و در نتیجه شادکامی کودکان بیمار مبتلا به سرطان استفاده می شود.

درمطالعه دینوری و همکاران (1394) به بررسی تأثیر نقّاشی و‌‌ موسیقی درمانی بر امید و ‌‌‌‌شادکامی کودکان 8 -11 سال بیمارستان محک پرداخته شده است. نمونه مورد مطالعه در این پژوهش 40 نفر از کودکان مبتلا به سرطان 8 - 11 سال بیمارستان محک شهر تهران بوده که با روش نمونه­برداری در دسترس انتخاب شدند. شرکت کنندگان به شیوه تصادفی انتخاب و به صورت تصادفی در 4 گروه شامل 3 گروه آزمایش و یک گروه کنترل جایگزین شده­اند. گروه­های آزمایش مداخله­های نقاشی به تنهایی، موسیقی درمانی به تنهایی و نقاشی همراه با موسیقی درمانی را دریافت کردند و گروه شاهد هیچ­گونه مداخله­ای دریافت نکرده­اند. هر 4 گروه در دومرحله زمانی پیش­آزمون و پس­آزمون توسط مقیاس شادکامی اکسفورد وامید کودکان اسنایدر ارزیابی شدند. نتایج آزمون تحلیل کوواریانس نشان داده است که مداخله روان شناختی (نقّاشی، موسیقی، تلفیقی) بر شادکامی در بین کودکان سرطانی تأثیر دارد از این رو اثر موسیقی درمانی، نقّاشی درمانی و موسیقی-نقّاشی درمانی با اطمینان 0.99 معنادار شده است. همچنین مداخله موسیقی-نقّاشی بر میزان امید در کودکان مبتلا به سرطان تأثیر داشته است ولی مداخله به تنهایی موسیقی و نقّاشی بر میزان امید در کودکان دارای سرطان تأثیر نداشته است .از یافته های این مطالعه جهت بهره جستن از هنر نقاشی برای افزایش شادکامی کودکان در محدوده سنی 7 تا 11 ساله مبتلا به سرطان استفاده شده است.

***نتایج مطالعات سایر نقاط دنیا:***

افزایش شادکامی به­واسطه بازی درمانی در گروه مورد مداخله در مقایسه با گروه کنترل در مطالعه گریپی[[6]](#footnote-5) و همکاران (2003) نیز که بر روی 11 کودک 3-5 سال دارای لوکمیا و بستری در بیمارستان کار کرده­اند، نشان داده شده است. شرکت کنندگان مبتلا به سرطان خون (11 نفر) از درمانگاه انکولوژی خارجی بیمارستان کودکان شهری بوده­اند. کودکان شاهد (11 نفر) یک مرکز مراقبت روزانه شرکت نموده­اند. موارد مورد بررسی شامل تجربه کودکان از استرس، رفتارهای بازی اجتماعی و شناختی، و خلق و خوی روزانه بوده است. نشان داده شده­است که کودکان مبتلا به لوسمی، در مقایسه با کودکان کنترل، پس از مداخله در یک رفتار بازی به­طور قابل توجهی افزایش شادکامی را داشته­اند. همبستگی پیرسون رابطه معناداری از شاد بودن و بازی برای کودکان مبتلا به لوسمی نشان داده است . بر اساس این مطالعه، می­توان چنین استنباط کرد که بازی درمانی حالا به اشکال مختلف برای کودکان تحت درمان بخصوص کودکان با شرایط ویژه (مبتلا به سرطان) ضروری به نظر می­رسد. این مطالعه نشان داده است که کودکان دارای لوکمیا با بازی درمانی توانسته­اند شادکامی خود را بیشتر کنند. همین که انتظار می­رود در مطالعه ما نیز کودکان دارای لوکمیا تعداد بیشتر آزمون دهنده­ها را شامل شوند، پس این طور پیش­بینی می­شود که در مطالعه ما نیز بتوانیم به نتایج مثبتی در زمینه استفاده از هنر و بازی برای افزایش شادکامی کودکان دست بیابیم. در پژوهش حاضر هم از نقش و کاربرد بازی درمانی (نقاشی بعنوان یکی از شاخه های اصلی و موثر بازی درمانی)در افزایش احساس شادی و شادکامی کودکان مبتلا به سرطان و تحت شیمی درمانی استفاده شده است.

نوریس و همکاران (2012) بررسی ارتباط بین فعالیت­های هنری و احساس مثبت- شادی در کودکان پیش دبستانی ( 3 و 4 و 5 ساله) را مورد بررسی قرار داده­اند. به هر یک از کودکان یک عروسک داده شده است و به آن­ها گفته شده که عروسک غمگین است و کودکان در صدد برمی­آمده­اند تا عروسک را خوشحال کنند. برای این کار کودکان به کشیدن نقاشی از فعالیتی که برای شاد کردن عروسک ( و در واقع خودشان) بوده است اقدام می­کرده­اند. در واقع اقدام خود را برای شاد کردن عروسک به صورت تصویر در می­آورده­اند. در این پژوهش هنر (نقاشی) تاثیر مثبتی بر افزایش احساسات مثبت نشان داده شده است . در مطالعه حاضر هم تلاش شده از طریق کشیدن نقاشی توسط کودکان سالم برای شاد کردن کودکان بیمار مبتلا به سرطان استفاده شود.

*در نهایت با توجه به نتایج مثبت وقابل توجه پژوهش های صورت گرفته در داخل و خارج کشور ،در پژوهش حاضر از کل مطالعات خارجی و داخلی از نقش همتایان در القا احساسات مثبت و شادی در کودکان همچنین از ابزار نقاشی بعنوان یکی از موثرترین شاخه های هنر در کودکان و از تعداد تقریبی حجم نمونه و محدوده سنی و در نهایت بررسی سطح شادکامی کودکان بیمار مبتلا به سرطان در رابطه با مداخلات هنری همچون نقاشی بهره جستیم.لازم به ذکر است که نوآوری این پژوهش مربوط به بررسی تمام موارد فوق بطور جامع در این تحقیق میباشد که در تمام تحقیق های انجام شده یکجا و کامل صورت نگرفته است و در این پژوهش سعی شده ارتباط تمامی عوامل ذکرشده با هم بررسی و نتایج ارزیابی و سپس جمع بندی شود.*

**هدف اصلي طرح^8^ :**

- تعیین تاثیر نقاشی همتایان بر شادکامی کودکان مبتلا به سرطان سنین مدرسه

1. **اهداف فرعي طرح^9^ :**

- تعیین و مقایسه میزان شادکامی كودكان قبل از مداخله نقاشی در گروه مداخله و کنترل
- تعیین و مقایسه میزان شادکامی كودكان بعد از مداخله نقاشی در گروه مداخله و کنترل
- مقایسه میزان شادکامی كودكان قبل و بعد از مداخله نقاشی در گروه مداخله و کنترل

1. اهدف كاربردي طرح^10^ :

با توجه به آن­که هنر در دنيای کودکان جايگاه ويژه و قابل توجهی داشته و معمولاً آنچه در سنين کودکی بر ذهن کودک نقش می­بندد تا سال­های دراز زندگی همراه اوست (بر سیر تکاملی وی تاثیر می­گذارد)، بررسی نقش هنر و به­ويژه نقاشی در فرآيند درمان و ساماندهی رفتارهای کودکانه اهميت بسزائی دارد. در حال حاضر نياز به درک صحيح جامعه­ی پرستاری کشور در نقش تأثيرگذار هنر در درمان پاره­ای بيماری­ها و اختلالات روانی، به­ويژه در کودکان و نيز درک اغلب متخصصان حوزه­ی درمان از نقش پررنگ هنر در راستای رفع ناهنجاری­های روانی از جمله مسائلي است كه اين پژوهش سعي دارد تا بر آنها اثر گذارد..

1. فرضيات^11^ يا سوالات پژوهش (باتوجه به اهداف طرح) :

نقاشی توسط همتایان بر شادکامی کودکان مبتلا به سرطان موثر است

1. نوع مطالعه را مشخص فرماييد و در قسمت روش اجرا موارد مقابل بند مورد اشاره را توضيح دهيد.

| محل علامت | نوع مطالعه | مواردي كه الزاما بايستي در روش اجراي طرح توضيح داده شود |
| --- | --- | --- |
|  | بررسي بيماران (Case series ) | تعريف بيماري - جمعيت مورد مطالعه - محلهاي مورد مطالعه |
|  | بررسي مقطعي(Cross sectional ) | جمعيت مورد مطالعه - نام متغيرهاي وابسته و مستقل - روشهاي نمونه‏گيري |
|  | مطالعه مورد/شاهد(Case / control ) | تعريف گروه بيماران و چگونگي انتخاب آنان - تعريف گروه كنترل و چگونگي انتخاب آنان - نسبت شاهد به مورد - نام متغير مستقل اصلي كه مورد بررسي قرار‏مي‏گيرد |
|  | مطالعه هم گروهي(Cohort) | بصورت آينده نگر (Prospective)يا گذشته نگر (Retrospective): تعريف جمعيت مورد مطالعه - تعريف دقيق مواجهه - تعريف دقيق Outcome - نحوه مقابله با Loss |
| ****** | مطالعه مداخله اي(interventional ) و يا كارآزمايي باليني (clinical trial ) | نوع مطالعه - نوع نمونه انساني يا حيواني - تعريف‏ نحوه مداخله و ميزان‏ دقيق‏آن (طول‏مدت‏,‏دوز مورد مصرف‏و …) - وجودگروه كنترل - نحوه تقسيم در گروه هاي مختلف (Allocation ) - نحوه كوركردن مطالعه - نحوه مقابله با خروج نمونه ها از مطالعه (Loss وwithdrawal ) - تعريف دقيق پيامد (‏outcome ) |
|  | مطالعات علوم پايه ( Experimental) | تعريف دقيق سير اجرا - تعريف دقيق بررسي نتايج |
|  | مطالعه براي ساخت دارو يا وسائل | تعريف دقيق دارو يا لوازم - آيا مشابه خارجي دارد در صورت وجودكاتالوگ آن پيوست باشد - موارد مصرف - نحوه تاييد دستگاه يا دارو |
|  | راه اندازي يك روش يا سيستم علمي/اجرايي |  |
|  | بررسي تست­ها | تعريف دقيق انجام تست - تعريف دقيق تست Gold standard - نحوه پذيرش بيماران وافراد سالم |
|  | بررسي روش­ها | مشخصات دقيق روش موردنظر - مشخصات دقيق روش مرسوم (routine ) -تعريف دقيق تفاوتها - نحوه پذيرش بيماران وافراد سالم |
|  | مطالعات كيفي | تعريف دقيق گروههاي مورد نظر – نحوه اجراي جلسات و هدايت بحثها - معرفي گردانندگان جلسات و تخصص آنها – نحوه نتيجه گيري |
|  | مطالعات مديريت سيستم بهداشتي | مشكل چيست؟ اطلاعات لازم براي بررسي مشكل كدامند؟ |
|  | طراحي نرم افزار | برنامه مورد استفاده براي طراحي – محتويات برنامه نرم افزاري – كاربردهاي نرم افزار |

**روش اجرا:**

پژوهش حاضر از نوع کارآزمایی بالینی تجربی، به­منظور بررسی تاثیر نقاشی بر شادکامی کودکان مدرسه ای (7 تا 11 ساله) تحت درمان سرطان می­باشد. پژوهشگر به منظور گردآوری داده­ها، پس از کسب اجازه از کمیته مشترک اخلاق دانشکده پرستاری و مامایی و دانشکده توانبخشی، و با ارائه معرفی نامه و کسب اجازه از مسئولین جهت انجام نمونه گیری به واحدهای پژوهش مراجعه خواهد کرد جامعه پژوهش شامل کودکان 7 تا 11 ساله مراجعه کننده برای درمان سرطان به بیمارستان17 شهریور رشت که دارای خصوصیات لازم برای انجام پژوهش هستند و معیارهای ورود به پژوهش را دارا می­باشند است. روش نمونه گیری دردسترس است. جهت جمع آوری اطلاعات از آزمون شادکامی کودکان استفاده خواهد شد. این تست شامل 20 سوال است و به صورت نمره دهی است. پرستار این سوالات را با زبان خود کودک به طوری که بتواند درک کند میپرسند و در پرسشنامه علامت میزنند.

**نوع پژوهش:**

پژوهش حاضر از نوع کارآزمایی بالینی می باشد. در این پژوهش اجرای برنامه مداخله نقاشی متغیر مستقل و شادکامی کودکان متغیر وابسته محسوب می­شوند.

**جامعه پژوهش:**

جامعه این پژوهش ، کلیه کودکان مراجعه کننده برای درمان سرطان (7 تا 11 ساله) به مرکز طبی کودکان هستند

محیط پژوهش:

محیط این پژوهش، بیمارستان مرکز طبی کودکان می­باشد.

**نمونه پژوهش:**

کلیه کودکان مبتلا به سرطان مراجعه کننده به مرکز مزبورکه دارای معیارهای ورود به مطالعه می باشند

**معیارهای ورود:**

کودک باید:

1. 11-7 سال، دارای پروندهدر بیمارستان باشد
2. نداشتن معلولیت ذهنی و جسمی مانع نقاشی کشیدن

کودکان همسالان مدرسه داوطلب شرکت در پژوهش

1. نداشتن معلولیت ذهنی و جسمی مانع نقاشی کشیدن
2. سن 11-7 سال

معیارهای خروج:

1. کودکانی که بیشتر از دو جلسه از کشیدن نقاشی خودداری کنند.
2. فوت کودک

روش محاسبه حجم نمونه و تعداد آن:

بمنظور برآورد حجم نمونه لازم در سطح معنی داری 5% و توان آزمون 80% و با فرض اينکه تاثیر نقاشی همتایان بر شادکامی کودکان مبتلا به سرطان سنین مدرسه

8/1نمره باشد (ده درصد حداکثر تمره ابزار)تا این تاثیر از نظر اماري معني دار تلقي گردد، پس از مقدار گذاري در فرمول:

n=
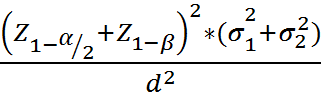


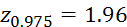


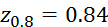


=10d

حجم نمونه درهرگروه 30 نفر محاسبه شد..

قابل ذکر است براساس دامنه تغییرات نمره شادکامی 0 تا 87می باشد ازفرمول =
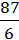

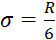
. انحراف معیار برابر14برآورد گردید. ضمنا با توجه به احتمال افت نمونه 10% به حجم نمونه فوق اضافه شد. لذا در نهايت حجم نمونه در هر گروه 33N= نفر تعيين گردید.

روش نمونه گیری

نمونه گیری به روش نمونه گیری دردسترس با تخصیص تصادفی بلوک بندی است شامل دو مرحله است . مرحله اول انتخاب نمونه ها که به روش در دسترس خواهدبود و مرحله دوم روش تخصیص نمونه ها به گروهها که به روش تصادفی بلوکی خواهد بود

روش تجزیه وتحلیل داده ها

ابتدا بااستفاده از آمارتوصیفی بروش تنظیم جداول توزیع فراوانی ومحاسبه شاخص های عددی نمونه ها توصیف خواهند شد. سپس با استفاده از آزمونهای کای دو وتی مستقل یکسانی وهمگونی متغیرها دردو گروه بررسی می شود. وسپس بااستفاده از ازآزمونهای تی مستقل وتی زوجی ودرصورت لزوم از آنالیز کوواریانس ، اهداف وآزمون فرضیه مورد تجزیه و تحلیل قرار خواهد گرفت.. تجزیه وتحلیل داده ها با استفاده ازنرم افزارspss نسخه 16 انجام خواهدشد

**نحوه مداخله (روش کار):**

در ابتدا محقق با اخذ اجازه از مسئولین مدرسه و هماهنگی با معلمین مربوطه، در جلسه ای با حضور والدین کودکان و با شرح اهداف و فرآیند تحقیق حاضر از آن­ها برای همکاری دعوت و فرم رضایت آگاهانه توسط ایشان تکمیل می­شود.سپس با هماهنگی با معلم مربوطه تعداد 33 دانش آموز برای اجرای نقاشی به شیوه نمونه­گیری در دسترس انتخاب می­شوند و با برنامه زمانی تعیین شده (هفتگی) و بمدت 5 جلسه و با قرار دادن ملزومات نقاشی(کاغذ A4،مداد رنگی،ماژیک های رنگی و مداد شمعی در رنگ های مختلف و به تعداد کافی) ، از آنها خواسته می شود تا برای کودکان بیمار مبتلا به سرطان که در بیمارستان در حال دریافت درمان هستند با هر موضوعی که خودشان خواستند(موضوع آزاد و اختیاری) نقاشی بکشند. کودکان توجیه می­شوند که از تمامی ابزارها و در رنگ­های مختلف، می­توانند به دلخواه استفاده نماید. چنانچه کودک تمایل به کشیدن بیش از یک نقاشی داشته باشد، برگه A4 اضافی در اختیار آنان خواهیم گذاشت. زمان نقاشی کردن کودک توسط محقق محدود نمی­شود و هر وقت کودک تمایل داشت نقاشی خود را به محقق ارائه می­دهد. تخمین زده می­شود؛ کودک در حدو20 دقیقه کار نقاشی خود را تمام کند، در غیر این صورت زمان بیشتری به آنها می­دهیم و در پایان جلسه نقاشی­های آنها جمع­آوری می­شود.سپس نقاشی ها هر هفته توسط محقق به بیمارستان برده میشود. در بیمارستان پس از هماهنگی با ریاست بیمارستان و مسئولین دفترپرستاری و بخش انکولوژی کار آغاز خواهد شد. نمونه های گروه مداخله و کنترل(هر گروه 33 نفر) بطور در دسترس نمونه گیری خواهند شد و به شیوه تصادفی بلوک بندی شده در هر گروه انتخاب میشوند. سپس فرم رضایت نامه شرکت در پژوهش از اولیا آنها اخذ می شود. همچنین پرسشنامه شادکامی قبل از انجام مداخله برای آنها با توضیح اهداف و فرآیند پژوهش تکمیل می گردد.بعد از تکمیل اولیه پرسشنامه شادکامی محقق از کودکان گروه مداخله میخواهد تا برای همتایان سالمشان که مشغول تحصیل در مدرسه هستند ، با موضوع کاملا اختیاری و آزادانه نقاشی بکشند.تعداد دفعات، مدت زمان و شرایط نقاشی در بیمارستان نیز مانند مدرسه است. تنها تفاوت در محیط نقاشی است که بچه­ها در کلاس درس و کودکان مبتلا به سرطان در بخشی که بستری می­باشند نقاشی را می کشند. سپس نقاشی کودکان بیمار هر هفته به کودکان مدرسه داده خواهد شد و از آنها درخواست میگردد. نقاشی بعدی را برای بچه های بیمار ترسیم کنند. و این چرخه به مدت 5 هفته ادامه خواهد یافت. در انتهای پنج هفته و پس از آنجام کامل مداخله، مجددا پرسشنامه شادکامی در کودکان بستری تکمیل خواهد شد.

**ابزار اندازه گیری:**

ابزار گرد آوری داده­ها در این پژوهش شامل: 1-پرسشنامه اطلاعات دموگرافیک؛ 2- فرم شادکامی کودکان

پرسشنامه اطلاعات دموگرافیک شامل: سن ، جنس، رتبه تولد ، میزان تحصیلات والدین، شغل پدر و مادر، تحصیلات کودک، وزن، سن شروع سرطان، مدت زمان انجام سرطان، بیماری­های زمینه­ای، نوع بیمه و عادت به نقاشی کردن است (سوالات این پرسشنامه در انتهای پروپوزال موجود می­باشد).

پرسشنامه شادکامی کودکان(راجرمورگان): مقیاس شادکامی کودکان که معادل انگلیسی آن The children`s Happiness Scalمی باشد دارای 20 گویه است و میزان شادکامی کودک را می سنجد که کودکان یا نوجوانان ممکن است راجع به خودشان مشخص کنند. این مقیاس توسط دکتر راجر مورگان )مدیر حقوق کودکان در انگلیس)و همکارانش از سال 2001 تا مارس 2014 با بررسی و کشف و گزارش نقطه نظرات و دیدگاه کودکان تحت مراقبت و همچنین دریافت کننده حمایت از بخش مراقبت اجتماعی و کودکانی که خارج از خانه در مدارس شبانه روزی ،کالج های آموزشی و سایر مراکز مراقبت از کودکان در سنین تا 13-14سال بودند ،جمع آوری و تدوین شده است. هر یک از موارد این مقیاس دیدگاه خود کودکان را ارایه می دهد بدون آنکه چیزی را که محقق، متخصصین، دولت یا افرادی که معمولا با آن موافق نیستند،کنار بگذارد.این گزارش شامل پرسشنامه ای میشود که دکتر مورگان و همکارانش برای بچه ها طرح کردند تا آن را پر کرده و به آنها بگویند چقدر احساس شادی می کنند. علاوه بر این،این مطالب نتایج استفاده از پرسشنامه را با بیش از دو هزار کودک و نوجوان در بر می گیرد.مقیاس شادکامی بر اساس دیدگاه ها و قضاوت های خود بچه هاست.دکترراجر مورگان و گروهش فهرستی از 100 نقطه نظر مطرح شده توسط بچه ها راجع به احساس شادی یا غمگینی را ارایه دادند. پس از آن از 147 کودک و نوجوان خواستند تا از این مقیاس درجه بندی برای قضاوت راجع به نحوه شادی یا غمگینی استفاده کنند. سپس آنها20 جمله ای که بچه ها در مورد قضاوت آنها از درجه بندی شادی مورد تایید قرار داده بودند را برداشت کردند و طیف خوبی از اظهارات از خیلی ناراضی(غمگین) تا خیلی شاد در مقایسه این نمره گذاری بدست آوردند.

روش نمره گذاری پرسشنامه شادکامی کودکان:این آزمون شامل 20 عبارت نمره بندی شده است.بالاترین نمره احتمالی (شادترین)25/4 است.نمره متوسط 88/2است (چیزی که محققان به آن میانه می گویند). پایین ترین نمره احتمالی 68/1 می باشد (در صورتی که هیچ یک از موارد پرسشنامه اصلا تیک زده نشده باشد). :این آزمون شامل 20 عبارت نمره گذاری شده است که کودکان یا نوجوانان ممکن است در مورد خودشان مشخص کنند. هر یک از مواردی که کودک راجع به خود قبول دارد تیک زده میشود سپس نمرات جمع میشود . جمع نمرات بدست آمده از عبارات بر تعداد آیتم های علامت زده شده تقسیم میشود.جواب به دست آمده نمره شادکامی کودک در این پرسشنامه می باشد.

پیامد مطالعه:

پیامد مورد بررسی "شادکامی " کودکان تحت درمان سرطان می­باشد.

1. ملاحظات اخلاقي^12^ :

پژوهشگر خود را موظف به رعایت اصول اخلاقی زیر دانسته است:

1. اخذ مجوز از کمیته مشترک اخلاق دانشکده پرستاری و مامایی و توانبخشی دانشگاه علوم پزشکی تهران
2. ارائه معرفی نامه و اخذ مجوز از مدیریت بیمارستان ها جهت انجام پژوهش
3. ارائه معرفی نامه به مسئولین محترم بخش ها و واحدهای مورد پژوهش
4. توضیح اهداف و ماهیت پژوهش برای کلیه واحدهای مورد پژوهش و رفع ابهامات آنان
5. اخذ رضایت آگاهانه کتبی و یا شفاهی از واحدهای مورد پژوهش و والدین آنها جهت شرکت در مطالعه
6. آزاد بودن واحدهای مورد پژوهش جهت شرکت در مطالعه یا خروج از آن
7. اطمینان دادن به واحدهای مورد پژوهش که عدم شرکت یا خروج آنها از مطالعه تاثیری بر روند بستری آنها جهت درمان سرطان در بیمارستان نخواهد گذاشت.
8. اطمینان دادن به واحدهای مورد پژوهش و والدین آنها نسبت به محرمانه بودن اطلاعات کسب شده
9. رعایت صداقت در نمونه گیری و جمع آوری و تجزیه و تحلیل داده ها
10. محدوديتهاي اجرايي طرح وروش كاهش آن­ها :

فعلا موردی بنظر نمی آید

جدول متغيرها :

| رديف | عنوان متغير | نوع متغير | | كمي | | كيفي | | تعريف علمي - عملي | نحوه اندازه گيري | مقياس |
| --- | --- | --- | --- | --- | --- | --- | --- | --- | --- | --- |
|  |  | مستقل | وابسته | پيوسته | گسسته | اسمي | رتبه‏اي |  |  |  |
| 1 | نقاشی |  |  |  |  |  |  | نقاشی عبارتست­از ترسیم خودانگیز تصاویر، که فرصت­هایی برای ارتباط و بیان غیرکلامی فراهم می­کند. نقاشی شیوه­ای مناسب در بیان عواطف و عقاید، نیازها و نابسامانی­های کودکان بوده و روش موثر در آموزش، تربیت و درمان آن­ها به شمار می­رود. کودک به کمک نقاشی، کشمکش­ها و دلهره­های درونی­اش را آشکار می­کند و به این ترتیب اثر آن­ها را کاهش می­دهد، در حقیقت وقتی مسائل و مشکلات بر روی کاغذ منتقل می­شوند، به صورت تازه و جداگانه­ای که کمتر دلهره­آور است در می­آیند . |  |  |
| 2 | شادکامی |  |  |  |  |  |  | شادي را ميزان و درجه­اي از مطلوبيت می­دانند كه انسان بر اساس آن كيفيت زندگي فردي خود را به عنوان يك كل ارزيابي می‌کند. به عبارت ديگر، مقدار علاقه فرد به زندگي شخصي خود، ميزان شادي فرد محسوب می‌شود. (74) | مقیاس شادکامی کودکان(دکتر راجر مورگان) | نمره |
|  |  |  |  |  |  |  |  |  |  |  |

1. پيش بيني كل زمان لازم براي اجراي كامل طرح به ماه :12 ماه
2. جدول زمان بندي مراحل اجراي طرح^13^

|  | | | | | زمان اجرا به ماه | | | | | | | | | | | | | | | | | | | | | | | | | | | | | | | | | | | | | | | | | | | | | | | | | | | | | | | | | | | | | | | | | | | | | | | |
| --- | --- | --- | --- | --- | --- | --- | --- | --- | --- | --- | --- | --- | --- | --- | --- | --- | --- | --- | --- | --- | --- | --- | --- | --- | --- | --- | --- | --- | --- | --- | --- | --- | --- | --- | --- | --- | --- | --- | --- | --- | --- | --- | --- | --- | --- | --- | --- | --- | --- | --- | --- | --- | --- | --- | --- | --- | --- | --- | --- | --- | --- | --- | --- | --- | --- | --- | --- | --- | --- | --- | --- | --- | --- | --- | --- | --- |
| رديف | فعاليتهاي اجرائي | | زمان كل | |  | |  | |  | |  | |  | |  | |  | |  | |  | |  | |  | |  | |  | |  | |  | |  | |  | |  | |  | |  | |  | |  | |  | |  | |  | |  | |  | |  | |  | |  | |  | |  | |  | |  | |  | |  | |
| 1 | مطالعات کتابخانه ای | | 3ماه | |  | |  | |  | |  | |  | |  | |  | |  | |  | |  | |  | |  | |  | |  | |  | |  | |  | |  | |  | |  | |  | |  | |  | |  | |  | |  | |  | |  | |  | |  | |  | |  | |  | |  | |  | |  | |
| 1. 22 | تدوین ابزار گردآوری اطلاعات تعیین روایی و پایایی ابزار | | 2 ماه | |  | |  | |  | |  | |  | |  | |  | |  | |  | |  | |  | |  | |  | |  | |  | |  | |  | |  | |  | |  | |  | |  | |  | |  | |  | |  | |  | |  | |  | |  | |  | |  | |  | |  | |  | |  | |
| 3 | جمع آوری اطلاعات از مراکز تعیین شده | | 6 ماه | |  | |  | |  | |  | |  | |  | |  | |  | |  | |  | |  | |  | |  | |  | |  | |  | |  | |  | |  | |  | |  | |  | |  | |  | |  | |  | |  | |  | |  | |  | |  | |  | |  | |  | |  | |  | |
| 1. 34 | تجزیه و تحلیل داده ها تهیه گزارش نهایی | | 1 ماه | |  | |  | |  | |  | |  | |  | |  | |  | |  | |  | |  | |  | |  | |  | |  | |  | |  | |  | |  | |  | |  | |  | |  | |  | |  | |  | |  | |  | |  | |  | |  | |  | |  | |  | |  | |  | |
| 1. 45 |  | |  | |  | |  | |  | |  | |  | |  | |  | |  | |  | |  | |  | |  | |  | |  | |  | |  | |  | |  | |  | |  | |  | |  | |  | |  | |  | |  | |  | |  | |  | |  | |  | |  | |  | |  | |  | |  | |
| 6 |  | |  | |  | |  | |  | |  | |  | |  | |  | |  | |  | |  | |  | |  | |  | |  | |  | |  | |  | |  | |  | |  | |  | |  | |  | |  | |  | |  | |  | |  | |  | |  | |  | |  | |  | |  | |  | |  | |
|  |  | |  | |  | |  | |  | |  | |  | |  | |  | |  | |  | |  | |  | |  | |  | |  | |  | |  | |  | |  | |  | |  | |  | |  | |  | |  | |  | |  | |  | |  | |  | |  | |  | |  | |  | |  | |  | |  | |
|  | | جمع | | 12 ماه | |  | |  | |  | |  | |  | |  | |  | |  | |  | |  | |  | |  | |  | |  | |  | |  | |  | |  | |  | |  | |  | |  | |  | |  | |  | |  | |  | |  | |  | |  | |  | |  | |  | |  | |  | |  |

توجه :

1. زمان طراحي پيش نويس طرح و تكميل اين فرم جزو زمان اجراي طرح محسوب نمي­شود.
2. دريافت گزارشها با توجه به جدول گانت مصوب صورت ميپذيرد. بنابر اين لازم است مجري طرح زمان ارائه گزارشات طرح را در اين جدول مشخص نمايد.
3. زمان شروع طرح بعد از تصويب آن، با هماهنگي مدير اجرايي طرح و حوزه مديريت امور پژوهشي و از هنگام تامين اعتبار در نظر گرفته ميشود.

## قسمت سوم – اطلاعات مربوط به هزينه‏ها

1. هزينه كارمندي (پرسنلي) باذكر مشخصات كامل و ميزان اشتغال هرفرد و حق الزحمه آنها :

| رديف | نوع فعاليت | نام فرد يا افراد | رتبه علمي | تعداد افراد | كل رقم حق الزحمه براي يك نفر | جمع كل |
| --- | --- | --- | --- | --- | --- | --- |
|  | **مدیر اجرائی طرح/ طراحی و design طرح پژوهشی- نظارت بر اجرای پژوهش** | **مجری طرح** |  |  |  | **5000000 ریال** |
|  | **استاد مشاور** |  |  |  |  | **1500000** |
|  | **استاد مشاور** |  |  |  |  | **1500000** |
|  | **استاد مشاور آمار** | **دکتر حقانی** |  |  |  | **3000000** |
|  | **استاد ناظر پایان نامه** |  |  | **دو نفر** |  | **1000000** |
|  | **پژوهشگر (نمونه گیری و جمع آوری اطلاعات)** |  |  |  |  | 1500000 |
|  | **همکار طرح ()** | **کمک پژوهشگر** |  | **یک نفر** |  | **1000000** |
|  | **وارد کردن اطلاعات و تجزیه و تحلیل یافته ها** |  |  |  |  |  |

1. هزينه آزمايشها وخدمات تخصصي كه توسط دانشگاه ويا ديگر موسسات صورت مي گيرد:

| موضوع آزمايش يا خدمات تخصصي | مركزسرويس دهنده | تعداد كل دفعات آزمايش | هزينه براي هر دفعه آزمايش | جمع ( ريال ) |
| --- | --- | --- | --- | --- |
|  |  |  |  |  |
|  |  |  |  |  |
| جمع هزينه هاي آزمايش­ها | | | | |

فهرست وسايل و موادي كه بايد از اعتبار اين طرح از داخل يا خارج كشور خريداري شود:

1. وسايل غيرمصرفي:

| نام دستگاه | كشورسازنده | شركت سازنده | شركت فروشنده ايراني | تعداد لازم | قيمت واحد | **قيمت كل** |
| --- | --- | --- | --- | --- | --- | --- |
|  |  |  |  |  |  |  |

1. موادمصرفي:

| نام ماده | كشورسازنده | شركت سازنده | شركت فروشنده ايراني | تعداديامقدار لازم | قيمت واحد | **قيمت كل** |
| --- | --- | --- | --- | --- | --- | --- |
| **کاغذ A4** | **ایران** |  |  | **4 بسته** |  |  |
| **آبرنگ** | **ایران** |  |  | **40 بسته** |  |  |
| **گواش** | **ایران** |  |  | **40 بسته** |  |  |
| **مدادرنگی** | **ایران** |  |  | **40 بسته** |  |  |
| **مدادشمعی** | **ایران** |  |  | **40 بسته** |  |  |
| **ماژیک** | **ایران** |  |  | **40 بسته** |  |  |
| **هدایا** | **ایران** |  |  | **40 عدد** |  |  |

هزينه مسافرت :

| مقصد | تعداد مسافرت در مدت اجراي طرح و منظور آن | نوع وسيله نقليه | تعداد افراد | هزينه به ريال |
| --- | --- | --- | --- | --- |
|  |  |  |  |  |
| جمع هزينه هاي مسافرت | | | | ريال |

هزينه هاي ديگر

| هزينه هاي تكثير اوراق | 100000 ريال |
| --- | --- |
| تایپ و ویراستاری | 100000 |
| هزينه هاي تكثير اوراق | 100000 ريال |
| ساير موارد ( پذیرایی از بیماران و والدین حین مداخله نقاشی: میوه هر نفر 20000 ریال ) | 500000 ريال |
| سایر فعالیتهای انگیزشی آموزشی در بیمارستانها (لوح یادبود به بیمارستانها جهت همکاری ) | 300000ريال |
| جمع کل هزینه های دیگر | 1100000 ريال |
|  |  |

جمع هزينه هاي طرح :

| هزينه پرسنلي | ............. ريال | هزينه مسافرت | ............. ريال |
| --- | --- | --- | --- |
| هزينه آزمايشها و خدمات تخصصي | ............. ريال | هزينه هاي ديگر | .............. ريال |
| هزينه مواد و وسايل مصرفي | ............ ريال |  | ............. ريال |
| هزينه وسايل غير مصرفي | ............ ريال | جمع كل | ............. ريال |

مبلغي كه از منابع ديگر كمك خواهد شد و نحوه مصرف آن : ………………………….ريال

باقيمانده هزينه هاي طرح كه تامين آن درخواست مي شود : ………………………….ريال

با مطالعه قسمت اول اين فرم و رعايت مفاد آن بدينوسيله صحت مطالب مندرج در پيش نويس را تائيد مينمايد واعلام ميداردكه اين تحقيق صرفا به صورت

1. يك طرح تحقيقاتي در دانشگاه علوم پزشكي تهران
2. بصورت مشترك با ……
3. در قالب پايان نامه در ……

ارائه شده است.

نام ونام خانوادگي

امضاي مجري يا مجريان طرح

#### فهرست منابع:

1. Van Rompay KK, Madhivanan P, Rafiq M, Krupp K, Chakrapani V, Selvam D. Empowering the people: Development of an HIV peer education model for low literacy rural communities in India. Human Resources for Health. 2008;6(1):1.

2. Redig AJ, McAllister SS. Breast cancer as a systemic disease: a view of metastasis: a view of metastasis. Journal of internal medicine. 2013;274(2):113-26.

3. Jafroodi M, Y. G. Epidemiologic evaluation of pediatric malignancies in 17 Shahrivar Hospital. Journal of Guilan University of Medecine Science

200; (68):14-21.

4. Mirzaie M YF, Navidi Z. . Survey personal and disease characteristics of children with cancer hospitalized in 17 shahrivar hospital, Rasht. Journal of Guilan Faculty of Medicine. 2010;19(61): 32-6.

5. Zareapour A, Falahi Khoshknab M, Kashaninia Z, Biglarian A, R. B. Effect of group play therapy on depression in children with cancer. . Scientific Journal of Kurdistan University of Medical Sciences. 2009; 14(3):64-72.

6. Mousavi SM, Pourfeizi A, Dastgiri S. Childhood cancer in Iran. Journal of pediatric hematology/oncology. 2010;32(5):376-82.

7. Hockenberry MJ, Wilson D, Wong DL. Wong's Essentials of Pediatric Nursing9: Wong's Essentials of Pediatric Nursing: Elsevier Health Sciences; 2012.

8. علیلو م, آباد هن, صفت فم. اثربخشی بازی درمانی براساس رویکرد لوی در کاهش اضطراب کودکان سرطانی. مجله پرستاری و مامایی جامع نگر. 2015;25(1):54-62.

9. Marlow DR, B. Text book of pediatric, 2010 nPWBSC.

10. Varni JW, Katz ER, Colegrove Jr R, Dolgin M. Perceived physical appearance and adjustment of children with newly diagnosed cancer: A path analytic model. Journal of Behavioral Medicine. 1995;18(3):261-78.

11. Larcombe I, Walker J, Charlton A, Meller S, Jones PM, Mott M. Impact of childhood cancer on return to normal schooling. BMJ. 1990;301(6744):169-71.

12. Susman EJ, Hollenbeck AR, Nannis ED, Strope BE, Hersh SP, Levine AS, et al. A prospective naturalistic study of the impact of an intensive medical treatment on the social behavior of child and adolescent cancer patients. Journal of Applied Developmental Psychology. 1981;2(1):29-47.

13. Jakobsson S, Ekman T, Ahlberg K, editors. Components that influence assessment and management of cancer-related symptoms: an interdisciplinary perspective. Oncology nursing forum; 2008.

14. Compas BE, Desjardins L, Vannatta K, Young-Saleme T, Rodriguez EM, Dunn M, et al. Children and adolescents coping with cancer: self-and parent reports of coping and anxiety/depression. Health Psychology. 2014;33(8):853.

15. Pinquart M, Teubert D. Academic, physical, and social functioning of children and adolescents with chronic physical illness: a meta-analysis. Journal of pediatric psychology. 2011:jsr106.

16. Alderfer MA, Hodges JA. Supporting siblings of children with cancer: A need for family–school partnerships. School mental health. 2010;2(2):72-81.

17. Michel G, Rebholz CE, Nicolas X, Bergstraesser E, Kuehni CE. Psychological distress in adult survivors of childhood cancer: the Swiss Childhood Cancer Survivor study. Journal of Clinical Oncology. 2010;28(10):1740-8.

18. Durualp E, Altay N. A Comparison of Emotional Indicators and Depressive Symptom Levels of School-Age Children With and Without Cancer. Journal of Pediatric Oncology Nursing. 2012;29(4):232-9.

19. Holder MD, Coleman B, Singh K. Temperament and happiness in children in India. Journal of Happiness Studies. 2012;13(2):261-74.

20. Gillespie Edwards A. Self Concept in Relationships and Learning, Caring for Childre

from Birth to Three. London: : National Children’s Bureau/PEEP; (2002).

21. Katz C, Hamama L. “Draw me everything that happened to you”: Exploring children's drawings of sexual abuse. Children and Youth Services Review. 2013;35(5):877-82.

22. Rollins JA. Tell me about it: drawing as a communication tool for children with cancer. Journal of Pediatric Oncology Nursing. 2005;22(4):203-21.

23. Councill T. Medical art therapy with children. Handbook of art therapy. 2003:207-19.

24. Nix GA, Ryan RM, Manly JB, Deci EL. Revitalization through self-regulation: The effects of autonomous and controlled motivation on happiness and vitality. Journal of Experimental Social Psychology. 1999;35(3):266-84.

25. Yost E, Ellis GD. Effect of Self Determination Theory-Based Recreation Activity-Staging on Vitality and Affinity Toward Nature Among Youth in a Residential Treatment Program. Residential Treatment for Children & Youth. 2008;23(1-2):5-26.

26. Vygotsky LS. Mind in society: The development of higher mental process. Cambridge, MA: Harvard University Press; 1978.

27. Tharp RG, Gallimore R. Rousing minds to life: Teaching, learning, and schooling in social context: Cambridge University Press; 1991.

28. Taghdisi M, NOORI SM, MERGHATI KE, Hoseini F, ASGHARNEJAD FA. Impact peer education approach on knowledge and practice about Mental Health of Adolescent Girls. 2012.

29. MOTEVASELIAN M, NASIRIANI K. Impact of Near-peer teaching on Learning Dressing Skill among Nursing Students. 2014.

30. Borzou R, Bayat Z, Salvati M, Homayounfar S. A comparison of Individual and Peer Educational Methods on Quality of life in patients with heart failure. Iranian Journal of Medical Education. 2014;14(9):767-76.

31. Thomas AM, Peterson L, Goldstein D. Problem solving and diabetes regiman adherence by children and adolescents with IDDM in social pressure situations: A reflection of normal development. Journal of Pediatric Psychology. 1997;22(4):541-61.

32. Shirazi M, Anoosheh M, Rajab A. The effect of self care program education by group discussion method on self concept in diabetic adolescent girls reffered to Iranian Diabetes Society. 2011.

33. Hamre HJ, Witt CM, Glockmann A, Ziegler R, Willich SN, Kiene H. Anthroposophic art therapy in chronic disease: a four-year prospective cohort study. Explore: The Journal of Science and Healing. 2007;3(4):365-71.

34. Jones EM, Landreth G. The efficacy of intensive individual play therapy for chronically ill children. International Journal of play therapy. 2002;11(1):117.

35. MAMIYANLOU H, ELHANI F, GHOFRANIPOUR F. Evaluation of the Effects of a Play Program on the Anxiety of Hospitalized School-Age Children in Tehran Medical Center of Children in 1999-2000. 2001.

36. دینور م, شمس اسفند آبادی ح, جلالی م. بررسی تاثیر نقاشی و موسیقی درمانی در شادکامی و امید کودکان سرطانی. تهران: دانشگاه بین‌المللی امام خمینی (ره); 1394.

37. نیکنامی, یعقوبی, یاسمن, پور ش, عطرکارروشن, نیا م. ‏ بررسی تأثیر بازی برمیزان اضطراب کودکان سن دبستان بستری در مرکز آموزشی-‏ درمانی کودکان گیلان. مجله پرستاری و مامایی جامع نگر. 2005;15(1):65-71.

38. Larigani B AF, Mohagery Tehrani MR, Tabatabaee A. . frequency Diabetics type 2 in Iran in year of 1380. Journal Diabetes & Lipid Iran ; . 1384; 4(3):75-82.

39. نادری ف, زينب اا. تاثير هنر درماني بر خودپنداره تاييدجويي و شادکامي کودکان مراجعه کننده به مراکز مشاوره و درمان شهرستان اهواز.

40. افسانه زپ, مسعود فخ, زهرا كن, اكبر ب, روناك ب. بررسي تاثير بازي درماني گروهي بر ميزان افسردگي كودكان مبتلا به سرطان.

41. Bolton P, Bass J, Betancourt T, Speelman L, Onyango G, Clougherty KF, et al. Interventions for depression symptoms among adolescent survivors of war and displacement in northern Uganda: a randomized controlled trial. Jama. 2007;298(5):519-27.

42. Noll RB. Evaluation of the school competency assessment scale: A critique. Journal of Pediatric Oncology Nursing. 2003;20(2):65-70.

43. López-Pérez B, Sánchez J, Gummerum M. Children’s and Adolescents’ Conceptions of Happiness. Journal of Happiness Studies. 2016;17(6):2431-55.

44. نعيمه ب, مهرنوش ن. اثر تکنيک هاي بازي درماني در کاهش اضطراب و افزايش احساسات مثبت و سطح سازگاري عمومي در کودکان 9-12 ساله مبتلا به سرطان خون.

45. Jangi S, Shirabadi A, Ansarhosein S, Ghoradel JA. Effecacy of painting therapy in reducing aggression in children with conduct disorder. Journal of Kermanshah University of Medical Sciences (J Kermanshah Univ Med Sci). 2014;18(8):443-51.

46. جنگی, شیرابادی, جانی, ستاره, پوراسمعلی. اثربخشی هنر‌درمانی بر پایه نقاشی‌ درمانی در کاهش اضطراب کودکان دچار لکنت زبان. مجله علمی پزوهشی دانشگاه علوم پزشکی ایلام. 2015;23(2):53-60.

47. فرامرزی, سالار, مرادی. تأثیر هنردرمانی با رویکرد نقاشی بر کاهش ناامیدی و تنهایی کودکان ناشنوای پسر. شنوایی شناسی-دانشگاه علوم پزشکی تهران. 2015;23(6):25-31.

48. پیریایی آ, پیریایی آ. تأثیر طراحی داخلی در بیمارستان کودکان سرطانی با رویکرد مدیریت استرس و تسریع بهبودی آن‌ها

بیمارستان فوق تخصصی کودکان محک تهران.

49. شیخذکریایی ن, کریدی گ, اردلان م. تاثير نقاشي بر اضطراب کودکان سرطاني بستري در بيمارستان.

50. ATTARI B, SHAFI AA, SALIMI H. The Effectiveness of Teaching Painting on Decreasing the Amount of Anxiety in Elementary School Boys. 2012.

51. Khodabakhshi Koolaee A, Vazifehdar R, Bahari F. Impact of painting therapy on aggression and anxiety of children with cancer. Caspian Journal of Pediatrics. 2016;2(2):135-41.

52. جهانگير ك, مصطفي ع, علي زي, كامران خ. اثربخشي نقاشي درماني در کاهش رفتارهاي پرخاشگرانه‌ دانش ‌آموزان دختر دچار نارساخواني.

53. نریمانی, بشرپور, سجاد, صومعه ع, سجاد. مقایسه عزت نفس و شادکامی در دانش آموزان ناشنوای مدارس تلفیقی و استثنایی. 2014.

54. Khadar MG, Babapour J, Sabourimoghaddam H. The effect of art therapy based on painting therapy in reducing symptoms of oppositional defiant disorder (ODD) in elementary School Boys. Procedia-Social and Behavioral Sciences. 2013;84:1872-8.

55. Khadar MG, Babapour J, Sabourimoghaddam H. The Effect of Art Therapy based on Painting Therapy in Reducing Symptoms of Separation Anxiety Disorder (SAD) in Elementary School Boys. Procedia - Social and Behavioral Sciences. 2013;84:1697-703.

56. خادر غ, باباپور, مقدم ص. اثربخشی نقاشی درمانی در کاهش نشانه های اختلال افسردگی کودکان دبستانی. پژوهش های روان‌شناسی بالینی و مشاوره. 2015;4(2):19-32.

57. Mousavi M, Sohrabi N. Effects of art therapy on anger and self-esteem in aggressive children. Procedia-Social and Behavioral Sciences. 2014;113:111-7.

58. Nezamipour EAA, Atefe%A Etemadinia, Mahin%A Ezadinia, Nasrin. The Efficacy of Drawing-Therapy on Reducing Aggressive Behavior of Hard Hearing Student. Journal of Exceptional Education. 2015;3(131):31-8.

59. Gariépy N, Howe N. The therapeutic power of play: examining the play of young children with leukaemia. Child: care, health and development. 2003;29(6):523-37.

60. Favara‐Scacco C, Smirne G, Schilirò G, Di Cataldo A. Art therapy as support for children with leukemia during painful procedures. Medical and pediatric oncology. 2001;36(4):474-80.

61. Barrera ME, Rykov MH, Doyle SL. The effects of interactive music therapy on hospitalized children with cancer: a pilot study. Psycho-oncology. 2002;11(5):379-88.

62. Sadruddin MM, Hameed-ur-Rehman M. Understanding the perceptions of children battling cancer about self and others through drawing. South Asian journal of cancer. 2013;2(3):113.

63. McCaffrey CN. Major stressors and their effects on the well-being of children with cancer. Journal of Pediatric Nursing. 2006;21(1):59-66.

64. de Mello Sabino MB, de Amorim Almeida F. Therapeutic play as a pain relief strategy for children with cancer. Einstein. 2006.

65. Holder MD, Coleman B. The contribution of temperament, popularity, and physical appearance to children’s happiness. Journal of Happiness Studies. 2008;9(2):279-302.

66. Holder MD, Coleman B, Wallace JM. Spirituality, religiousness, and happiness in children aged 8–12 years. Journal of Happiness Studies. 2010;11:131-50.

67. Norris A. Children and Art: Exploring the Correlation between Art Activities and Positive Emotion/Happiness in Preschoolers. 2012.

68. Puetz TW, Morley CA, Herring MP. Effects of creative arts therapies on psychological symptoms and quality of life in patients with cancer. JAMA internal medicine. 2013;173(11):960-9.

69. Tsai Y-L, Tsai S-C, Yen S-H, Huang K-L, Mu P-F, Liou H-C, et al. Efficacy of therapeutic play for pediatric brain tumor patients during external beam radiotherapy. Child's Nervous System. 2013;29(7):1123-9.

70. Giacomoni CH, Souza LKd, Hutz CS. O conceito de felicidade em crianças. Psico USF. 2014;19(1):143-53.

71. Brown J. Group medical play for reducing stress and improving mood in children going to visit the pediatrician: The University of Alabama TUSCALOOSA; 2012.

72. do Vale Pinheiro I, da Costa AG, Rodrigues DCB, de Paula Oliveira N, Malheiro A, Ramos JL. Hospital psychological assessment with the drawing of the human figure: A contribution to the care to oncologic children and teenagers. Psychology. 2015;6(04):484.

73. به‌پژوه, نوری. تأثیر نقاشی درمانی در کاهش رفتارهای پرخاشگرانه دانش آموزان عقب مانده ذهنی. روانشناسی و علوم تربیتی (دانشگاه تهران). 2002;2(32):155-70.

74. Dr Roger Morgan OBE ,The children's happiness scale ,March 2014

.75 Cartagena RG, Veugelers PJ, Kipp W, Magigav K, Laing LM, Effectiveness of an HIV prevention program for secondary school students in Mongolia . Journal of Adolescent Health . 2006; 39(6): 925. e9-e16

.76-اصفهانی،زهرا-رضازاده ،مجید-سیگاری،سپیده-فرهودی،بهنام-احترامی،مهرداد-شیبانی،شهناز و دیگران(1389)آموزش پیشگیری از ایدز برای افراد در معرض خطر.تهران،ایران:دانشگاه علوم پزشکی بقیه ا...(عج)،مرکز تحقیقات علوم رفتاری.

1. Global Symptom Index [↑](#footnote-ref-2)
2. Barbara Fredrickson [↑](#footnote-ref-3)
3. Malchiodi [↑](#footnote-ref-4)
4. [↑](#endnote-ref-2)
5. **رضايت نامه شرکت در طرح تاثیر نقاشی همتایان بر شادکامی کودکان مبتلا به سرطان سنین مدرسه**

   **آقاي/ خانم محترم**

   بدين وسيله از شما جهت شركت در پژوهش فوق‌الذکر دعوت به عمل مي‌آيد. اطلاعات مربوط به این پژوهش در این برگه خدمتتان ارائه شده است و شما برای شرکت یا عدم شرکت در این پژوهش آزاد هستید.

   شما مجبور به تصميم گيري فوري نيستيد و برای تصميم گيري در اين باره مي‌توانيد سوالات خود را از تيم پژوهشي بپرسيد و با هر فردي که مایل باشید مشورت نماييد. قبل از امضاي اين رضايت نامه مطمئن شويد كه متوجه تمامي اطلاعات اين فرم شده‌ايد و به تمام سوالات شما پاسخ داده شده است.

   **مجري پژوهش**

   مي‌دانم كه اهداف اين پژوهش عبارتند از:

   افزایش شادکامی کودک من

   1. من مي­دانم که شرکت من در اين پژوهش کاملاً داوطلبانه است و مجبور به شرکت در اين پژوهش نيستم.

   به من اطمينان داده شد که اگر حاضر به شركت در اين پژوهش نباشم، از مراقبت‌هاي معمول تشخيصي و درماني محروم نخواهم شد و رابطه درماني من با مركز درماني و پزشك معالجم دچار اشكال نمي‌شود.

   1. من مي‌دانم كه حتي پس از موافقت با شركت در پژوهش مي‌توانم هر وقت كه بخواهم، پس از اطلاع به مجري، از پژوهش خارج شوم و خروج من از پژوهش باعث محرومیت از دریافت خدمات درمانی معمول برای من نخواهد شد.
   2. نحوه‌ي همکاري اينجانب در اين پژوهش به اين‌صورت است:
   3. کودک من هفته ای یک نقاشی برای محقق می کشد و نقاشی کودکان دیگر را هم تکمیل می کند
   4. منافع احتمالي شرکت اينجانب در اين مطالعه به اين شرح است:

   افزایش شادکامی

   1. آسيب‌ها و عوارض احتمالي شرکت در اين مطالعه به اين شرح است:

   ندارد

   1. در صورت عدم تمایل به شرکت در مطالعه روش معمول درمانی برای من ارائه خواهد شد که منافع و عوارض آن به این شرح است:

   ندارد

   1. من مي­دانم كه دست اندر كاران اين پژوهش، كليه اطلاعات مربوط به من را نزد خود به صورت محرمانه نگه‌داشته و فقط اجازه دارند فقط نتايج كلي و گروهي اين پژوهش را بدون ذکر نام و مشخصات اينجانب منتشر كنند.
   2. می­دانم که كميته اخلاق در پژوهش با هدف نظارت بر رعایت حقوق اينجانب مي‌تواند به اطلاعات من دسترسي داشته باشد.
   3. من مي‌دانم كه هيچ‌يک از هزينه‌هاي انجام مداخلات پژوهشي به شرح ذيل بر عهده من نخواهد بود.
   4. خانم / آقاي خدیجه زارعیجهت پاسخگويي به اينجانب معرفي شد و به من گفته شد تا هر وقت مشكلي يا سوالي در رابطه با شركت در پژوهش مذكور پيش آمد با ايشان در ميان بگذارم و راهنمايي بخواهم.

   آدرس و شماره تلفن ثابت و همراه ايشان به شرح به من ارائه شد:

   - **آدرس: میدان توحید- دانشکده پرستاری و مامایی علوم پزشکی تهران- ساختمان شماره 2 اتاق 421**
   - **تلفن ثابت: 61054421**
   - **تلفن همراه: 09123212190**
   1. من مي‌دانم كه اگر در حين و بعد از انجام پژوهش هر مشكلي اعم از جسمي و روحي به علت شرکت در اين پژوهش براي من پيش آمد درمان عوارض، و هزينه‌هاي آن و غرامت مربوطه بر عهده مجري خواهد بود.
   2. من مي­دانم اگر اشکال يا اعتراضي نسبت به دست اندركاران يا روند پژوهش دارم مي­توانم با كميته اخلاق در پژوهش دانشگاه علوم پزشكي تهران به آدرس: **تهران، تقاطع بلوار كشاورز و خيابان قدس، ساختمان ستاد مركزي دانشگاه علوم پزشكي تهران، طبقه ششم، مديريت امور تحقيقات و فناوري، دبيرخانه كميته اخلاق در پژوهش دانشگاه، تلفن 81633626 و 81633613 تماس** گرفته و مشکل خود را به صورت شفاهي يا كتبي مطرح نمايم.
   3. اين فرم اطلاعات و رضايت آگاهانه در دو نسخه تنظيم شده و پس از امضا يک نسخه در اختيار من و نسخه ديگر در اختيار مجري قرار خواهد گرفت.

   اينجانب موارد فوق‌الذکر را خواندم و فهميدم و بر اساس آن رضايت آگاهانه خود را براي شركت در اين پژوهش اعلام مي‌کنم.

   امضاي شركت كننده

   اينجانب خدیجه زارعیخود را ملزم به اجراي تعهدات مربوط به مجري در مفاد فوق دانسته و متعهد مي‌گردم در تأمين حقوق و ايمني شركت كننده در اين پژوهش تلاش نمايم.

   مهر و امضاي مجري پژوهش

   **رضايت نامه شرکت در طرح تاثیر نقاشی همتایان بر شادکامی کودکان مبتلا به سرطان سنین مدرسه**

   **آقاي/ خانم محترم**

   بدين وسيله از شما جهت شركت در پژوهش فوق‌الذکر دعوت به عمل مي‌آيد. اطلاعات مربوط به این پژوهش در این برگه خدمتتان ارائه شده است و شما برای شرکت یا عدم شرکت در این پژوهش آزاد هستید.

   شما مجبور به تصميم گيري فوري نيستيد و برای تصميم گيري در اين باره مي‌توانيد سوالات خود را از تيم پژوهشي بپرسيد و با هر فردي که مایل باشید مشورت نماييد. قبل از امضاي اين رضايت نامه مطمئن شويد كه متوجه تمامي اطلاعات اين فرم شده‌ايد و به تمام سوالات شما پاسخ داده شده است.

   **مجري پژوهش**

   1. من مي‌دانم كه اهداف اين پژوهش عبارتند از:

   افزایش شادکامی کودکان بیمار مبتلا به سرطان

   1. من مي­دانم که شرکت فرزند من در اين پژوهش کاملاً داوطلبانه است و مجبور به شرکت در اين پژوهش نيستم.

   به من اطمينان داده شد که اگر حاضر به شركت فرزندم در اين پژوهش نباشم، از روند معمول تحصیلی محروم نخواهد شد و رابطه کودک من با مدرسه و اولیا مدرسه دچار اشكال نمي‌شود.

   1. من مي‌دانم كه حتي پس از موافقت با شركت در پژوهش مي‌توانم هر وقت كه بخواهم، پس از اطلاع به مجري، کودک خود را از پژوهش خارج نمایم و خروج فرزند من از پژوهش باعث محرومیت از دریافت خدمات تحصیلی معمول برای وی نخواهد شد.
   2. نحوه‌ي همکاري فرزند اينجانب در اين پژوهش به اين‌صورت است:
   3. کودک من هفته ای یک نقاشی با موضوع آزاد برای کودکان همسال مبتلا به سرطان می کشد(به تعداد 5 نقاشی)
   4. منافع احتمالي شرکت در اين مطالعه به اين شرح است:

   افزایش شادکامی

   1. آسيب‌ها و عوارض احتمالي شرکت در اين مطالعه به اين شرح است:

   ندارد

   1. در صورت عدم تمایل به شرکت در مطالعه، روش معمول تحصیلی برای کودک من ارائه خواهد شد که عوارض آن به این شرح است:

   ندارد

   1. من مي­دانم كه دست اندر كاران اين پژوهش، كليه اطلاعات مربوط به کودک من را نزد خود به صورت محرمانه نگه‌داشته و فقط اجازه دارند فقط نتايج كلي و گروهي اين پژوهش را بدون ذکر نام و مشخصات اينجانب منتشر كنند.
   2. می­دانم که كميته اخلاق در پژوهش با هدف نظارت بر رعایت حقوق کودک اینجانب مي‌تواند به اطلاعات فرزندم دسترسي داشته باشد.
   3. من مي‌دانم كه هيچ‌يک از هزينه‌هاي انجام مداخلات پژوهشي به شرح ذيل بر عهده من نخواهد بود.
   4. خانم / آقاي خدیجه زارعی جهت پاسخگويي به اينجانب معرفي شد و به من گفته شد تا هر وقت مشكلي يا سوالي در رابطه با شركت در پژوهش مذكور پيش آمد با ايشان در ميان بگذارم و راهنمايي بخواهم.

   آدرس و شماره تلفن ثابت و همراه ايشان به شرح به من ارائه شد:

   - **آدرس: میدان توحید- دانشکده پرستاری و مامایی علوم پزشکی تهران- ساختمان شماره 2 اتاق 421**
   - **تلفن ثابت: 61054421**
   - **تلفن همراه: 09123212190**
   1. من مي‌دانم كه اگر در حين و بعد از انجام پژوهش هر مشكلي اعم از جسمي و روحي به علت شرکت در اين پژوهش براي کودک من پيش آمد درمان عوارض، و هزينه‌هاي آن و غرامت مربوطه بر عهده مجري خواهد بود.
   2. من مي­دانم اگر اشکال يا اعتراضي نسبت به دست اندركاران يا روند پژوهش دارم مي­توانم با كميته اخلاق در پژوهش دانشگاه علوم پزشكي تهران به آدرس: **تهران، تقاطع بلوار كشاورز و خيابان قدس، ساختمان ستاد مركزي دانشگاه علوم پزشكي تهران، طبقه ششم، مديريت امور تحقيقات و فناوري، دبيرخانه كميته اخلاق در پژوهش دانشگاه، تلفن 81633626 و 81633613 تماس** گرفته و مشکل خود را به صورت شفاهي يا كتبي مطرح نمايم.
   3. اين فرم اطلاعات و رضايت آگاهانه در دو نسخه تنظيم شده و پس از امضا يک نسخه در اختيار من و نسخه ديگر در اختيار مجري قرار خواهد گرفت.

   اينجانب موارد فوق‌الذکر را خواندم و فهميدم و بر اساس آن رضايت آگاهانه خود را براي شركت در اين پژوهش اعلام مي‌کنم.

   امضاي شركت كننده

   اينجانب خدیجه زارعیخود را ملزم به اجراي تعهدات مربوط به مجري در مفاد فوق دانسته و متعهد مي‌گردم در تأمين حقوق و ايمني شركت كننده در اين پژوهش تلاش نمايم.

   مهر و امضاي مجري پژوهش

   **The Children’s Happiness Scale**

   مقیاس شادکامی کودکان(دکتر راجر مورگان):

   زندگی تا الان برای من خوب بوده است. 3.64

   من منطقی رفتار می کنم 3.13

   می دانم در آینده چه اتفاقی در زندگی ام می افتد 3.15

   مشکلات بزرگی دارم اما آنها را حل و فصل میکنم 2.55

   به خودم کلا افتخار می کنم 3.65

   سعی میکنم بعضی چیزها را در خودم تغییر بدهم 2.57

   هیچ مشکل بزرگی تا الان ندارم 3.22

   دوستان زیادی دارم 4.01

   از چیزی که پیش آمده گیج شدم 2.43

   هرگز احساس امنیت نکردم 1.74

   اغلب مضطرب می شوم 2.32

   احساس تنهایی می کنم 1.68

   مردم نسبت به من مغرضانه عمل می کنند 1.77

   از اشتباهاتم درس می گیرم 3.18

   خجالتی هستم 2.63

   اذیت میشوم 1.68

   چیزهای جدید را خوب یاد می گیرم 3.70

   هنگامی که به کمک نیاز دارم همه به من کمک میکنند 3.38

   سرگرمی های زیادی دارم 4.25

   - به راحتی غمگین می شوم 1.75

   [↑](#endnote-ref-3)
6. Gariepy [↑](#footnote-ref-5)
