# Supplementary material for: The effect of exchanging drawings with peers on the happiness of children with cancer, aged 7–11 years: A clinical trial
Source: PLoS One. 2021 Oct 15;16(10):e0257867. doi: 10.1371/journal.pone.0257867 (PMC8519419; doi:10.1371/journal.pone.0257867)
Supplement: S2 File — (DOCX) [file pone.0257867.s005.docx]

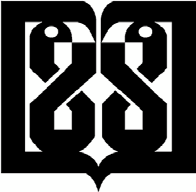


Tehran University of Medical Sciences

**Office of TUMS-IC Vice President for Research Affairs**

Title:

The effect of painting by peers on happiness in school-aged children with cancer

**Note:** This form is provided to you in **Word format** to make it easy for you to enter information directly into the document; however you are not allowed to change or alter items or format of this form in anyways.

- If any part of the form does not apply to you, put N/A in that part instead of deleting it.

**Failure to comply with this paragraph will result in rejection of your proposal or a significant delay in processing it.**

**Research Title:**

The effect of painting by peers on happiness in school-aged children with cancer

**Full Name of the Project Manager(s):**

Dr. A. Sadat Hosseini

**School/Research Center:**

Tehran University of Medical Sciences, school of Nursing and Midwifery

**Type of Research:**

Basic Applied/Clinical HSR

**This research is a:**

Student Thesis ▄ ▄

TUMS Research Project **

Joint Research Project

**Project Description**

***If a thesis, specify the level:*** Undergraduate M.Sc*. MPH Ph.D. Postdoc.

*

**Type of Study: Please mark**

| Case series |  |
| --- | --- |
| Cross-sectional |  |
| Case / control |  |
| Cohort |  |
| / interventional clinical trial |  |
| Experimental |  |
| Pharmaceutical Study |  |
| Implementation of a scientific/ executive Method |  |
| Test Review |  |
| Method Review |  |
| Qualitative | ▄ |
| Health System Management Study |  |
| Software Design |  |

**Information about the Project Manager(s)**

- Full Name(s): Dr. A. Sadat Hosseini
- Academic Rank: Associate Professor
- School/Research Center: Tehran University of Medical Sciences
- Department: Pediatric
- Research Location: Iran
- Expected Duration: 12 months
- Current Position and work location: Associate Professor
- Work Phone Number: 02161054423
- Work Address: Nosrat, Tohid Street
- E-mail Address: ashoseini@tums.ac.ir
- **Contact Number in case of emergency: 0989122094828**

**Research Project Team:** (Other supervisors, advisors, students, other partners)

|  | Full Name | Position and Academic Rank | Type of Involvement | E-mail address and Phone Number | Partner’s Signature |
| --- | --- | --- | --- | --- | --- |
| 1 | A. Sadat Hosseini | Associate Professor | Supervisor | ashoseini@tums.ac.ir |  |
| 2 | Khadigeh Zaree |  | Advisor |  |  |
| 3 | Somayeh Palvan | Student | Data collection |  |  |
| 4 |  |  |  |  |  |
| 5 |  |  |  |  |  |
| 6 |  |  |  |  |  |
| 7 |  |  |  |  |  |
| 8 |  |  |  |  |  |
| 9 |  |  |  |  |  |
| 10 |  |  |  |  |  |

| **1- Proposal Abstract (max 300 words):**  **Introduction:** Today due to the increasing progress in early diagnosis, advanced laboratory technology, effective and successful treatment and pathology, children with cancer are able to live longer. This disease not only threatens the child's health as a tense factor, but also is considered as a factor threatening mental health that effects the life of the patient in all emotional, mental, social, cultural, and affective contexts. In this case, completing intervention such as using art in children with cancer, can increase their ability in relation to issues of the disease, and encourages the patient to gain the potential to control their situation. Painting is considered as one of the methods of using art to improve children's mental health. Research has shown that we can increase happiness in children with cancer by using painting and holding classes. By using this method, we can make up for children's not going to school and also improve their self-confidence and make them happy. In this way, the child can see themselves in a completely free place and as a result the treatment process will work better. In school age children, peers have a significant role in mental evolution and promotion of psychological situation, and are considered as an available and effective potential that enable children to help each other. Therefore, the aim of this study is to assess the effect of painting with peers on children with cancer's happiness in the school age. If the findings of this study are effective, painting, can be used as a nursery intervention to improve the health of children who are hospitalized. On the other hand, peer children can be used as a passionate and practical power to improve the psychological situation of children.  **Method:** This study is a clinical trial that examines the effect of independent variable (intervention of drawing with peers) on dependent variable (happiness) in children with cancer. 33 children aged 7 to 11, studying in school as peer group, 66 children with cancer (using randomize selection, 33 children with cancer in intervention group and 33 children in control group) are chosen. Well informed and based on criteria for children with cancer participation they should: 1. Be at age ranged from 7-11 and have a file in the hospital; 2. Can understand and answer questions; not have a physical disability in order to be able to draw pictures. Criteria for exclusion: 1. Children who are not able to participate in drawing classes. 2. Child's death  Participation criteria for peers: 1. Aged 7-11, 2. studying at school and 3. Not having a disability for drawing.  Place of study: Central Hospital of Children. To start intervention, first the peers are provided with tools needed for painting such as paper, colored pencils, crayons, erasers and they are asked to draw for their ill peers about a free subject and in an unlimited time. This process is done for 5 sessions and there are one week intervals between the sessions. Then the drawings are given to the ill children by the researcher and they are asked to draw for their healthy peers about any desired subject. There are 5 sessions for each group (5 sessions for the peer group and 5 sessions for the intervention and control group) and there are one week intervals between them. The situation for drawing and their tools are the same as peer children at school. For the control group, the routines are done too. A questioner will be completed by the children in both groups (control and intervention), once in the beginning of the study and once at the end of the fifth week. Children's happiness will be assessed based on their changes before and after the intervention for both groups. The questionnaire will be examined by the researcher; then using SPSS, the relationship between them will be examined and statistic tests, such as covariance and t-test, will be used to assess the variables.  **Key Words:** painting- happiness-peers-cancer-nursing |
| --- |

| **2- Rationale and Backgrounds:** Despite advances in the diagnosis and treatment of malignancies, cancer is the second cause of death in children (2). During different years in our country, the number of children with cancer has increased from nine children per 100,000 children per year to 15 children in 2008 (3). Considering the fact that any changes and illnesses in the child threatens his health, it will cause the child's needs to not be met (4). Cancer has deep psychological effects on children. On the other hand, hospitalization and being in the sad and soulless environment of the hospital upsets them. Also, long-term and painful treatments of this disease increase the current mental problems (5). Hospitalization for children is a crisis in adapting to the conditions of the new environment, in a situation where they are not only unhealthy but also exposed to unknown and dangerous factors. Thus, hospitalization of the child causes anxiety resulted from separation, grief, fear of the new environment and fear of disability (6). many studies have shown that younger people with cancer who are exposed to chemotherapy are exposed to the risk of mental problems are in the two areas of social adjustment with peers and feeling of being good (7-9) most of the time illness and hospitalization is the first crisis that children encounter (10). Statistical reports have shown that 50-80 percent patients with suffer from mental health problems at the same time. It has been shown that children that rescued from cancer had psychological problems that, according to the Global Symptom index of sensitivity to mutual relationships, their depression and violence, have increased (12). Therefore, it is necessary to use methods that minimize the rate of these complications and problems. Since children know themselves by making connections with others, their opinions and beliefs about themselves are influenced by the opinions of people around them. Children often know through the opinions of others that they are a good person or not. Are they lovely or not? Children who have a good feeling about themselves have developed a positive self-concept in themselves; And in comparison to other children, they communicate better with others, they are happy people and they think of being successful (14). Children's asense of self-worth improves when they understand that close people who are in contact with them, pay attention to them, think about them, and enjoy being with them. And they want to be near them. (14) In order to solve children's problems, we should talk to them using their language and in their own way to get the desired effect, and art is one of the methods of speaking in children. (15) using art as a method of treatment of psychological disorders has attracted the attention of researchers; Many researchers, psychologists and specialists in the relevant majors from the distant past to the present have been searching for using art as a tool for curing mentally and internally in addition to conventional physical and mental therapies, that results in improving the body. Art as an aesthetic phenomenon has a particular power in such a way that it can improve the conflicting forces within and between individuals and create a better adaptation for the owner of the using of art itself includes a wide range of functions of art elements such as theater, painting, music and color, and on one side of this range, art as a means of non-verbal communication is effective, and on the other hand It is a tool to improve the psychological state of children (18,19). Many studies have been done in relation to the power of art, and research shows that one of the most important arts that is used in children, which can be very useful is the visual arts. One of the branches of visual arts is painting (17,20). 10) (10) In other words painting provides a chance for children who are not able to express themselves in by using words a natural way. (13). Painting is a tool for expressing children's emotions; This means that children reflect their insights and internal worlds through pictures. Children's paintings are the purest manifestation of their beautiful childish nature and are therefore as meaningful and valuable as the children themselves. With each of their works, they express a part of their being. (21) considering the above mentioned facts, it is clear that one can use art to improve the psychological condition of children. One of the problems of the children who are hospitalized is grief caused by hospitalization and being apart from friends and school. Therefore, improving the psychological condition of children in this dimension is a problem that has been forgotten up to now. One of the ways of improving the psychological condition of children is to consider the happiness of the hospitalized children. Barbara Frederickson states that positive emotions have an important role in human development. Positive emotional thoughts provide a wide range of thoughts, movements, games, explorations, and creativity in the individual. (22) This issue shows the role of happiness in the growth and development of the child. Therefore, by using art, we can bring this growth and development to the children who are hospitalized. In Malchiyudi (2003) study, creative arts such as painting and making sculpture with soil were used and based on the observations, he realized that artistic creativity results in hope, self-esteem, independence, sense of competition and showing emotions in ill children. He states that in the process of using art in children with diseases, children can convey their perceptions, needs and desires. Finally, he points out that by using art we can accustom the children to the hospital environment and displaying their works of art we can make a sense of self-confidence, acceptance, security and friendship in them (15). And also we strengthen the sense of vitality, optimism and hope among these children. Vitality and excitement, that are known as the feeling of passion, freshness and full of energy, are important psychological factors that promotes the health of chronic patients and reduce the risk of mental problems (16). In fact, using art results in problem solving, and expression of emotion, and this happy ending is always accompanied by happiness, cheerfulness, and a sense of joy in children. (25).  One of the types of education for patients is peer education, which has a great effect on education and development of health and creating an environment for learning. The exchange of information, insights and behaviors is done by people who are not educated in it in a special way (17). The effectiveness of the peer education approach is based on the theory that sensitive information can be shared between peers more easily. Of the advantages of peer group, we can name things such as improving social norms, supportive, positive attitudes and healthy behaviors, peers' good knowledge of the target group's social and cultural environment, and being involved in painting their desired projects (18). By promoting empathy and trust, peers are also a strong link between the health system and other peers) (19). Considering the importance of having communication with peers in childhood, peer support can be considered as a potential goal in therapeutic or care interferences (20). Childhood is a period of being influenced by peers and imitating them, and peer, as an available model, has effects on people of the same age. In this study, by holding painting classes by peers, maybe we can fill the gap in not going to school in them in a way that this feeling is strengthened in them that they are still in class and the experience of being in class and communicating with their peers. On the other hand, the nurse, by being with the child's personality properties, can help in the process of recovery of the child under treatment (21, 22). Therefore, peer as an available element and eager to help can be very effective in improving the psychological condition of children. Considering all that is said, it can be concluded that the using of peers in the form of painting as a way of using art to promote the health of children may improve the psychological status and happiness of children with cancer, so this study seeks to examine the effect of painting with peers on the happiness of children with cancer.  **Literature review**  **Research which are done in Iran**  In order to review the text in this study, various databases such as Pubmed, Scopus, Web of Science, and Scholar were examined. Search for the keywords 'Happiness', 'painting', 'peers', 'school-aged children' and 'cancer' is done first in the title, abstract and keywords of these databases in English without considering the time period. Also searching for, keywords (happiness, painting, peers, school-aged children and cancer) was done in domestic databases including Iranmedex, Magiran, SID, Scholar and Google in Persian and without considering the time period.  So far, art therapy has been performed for many diseases such as chronic patients (33), for children with diabetes (34) and for children hospitalized in Tehran Children's Medical Center (35) and significant results have been observed.  **Researches which are done in Iran:**  Today, the comparison of care, especially the peer-centered care model, is considered. A review study was done by Kazemi et al. (2013) which aimed to assess the effectiveness of peer-centered intervention in the management of type 1 diabetes in children and adolescents by using different databases. The criteria for using studies that are done, in this systematic review has included peer-centered articles about type 1 diabetes in children and adolescents under the age of 20. The results have shown that peer-centered interferences can have a positive effect (23). According to findings of this study peers and friends' participation can be used as an effective method in educating the patient. In addition, peer education and participation has a wide range and dimensions that nurses and teachers can use to ensure and promote the health of children and adolescents with diabetes, as well as other hospitalized children including children with cancer. The positive effect of peer interaction and participation in this peer-centered research is used to reach the goals of the current study. Therefore, in creating and increasing children's happiness, the very effective power of peer is used.  Naderi et al. (2009) have investigated the effect of art therapy on self-concept of approval and happiness of children referred to counseling and treatment centers in Ahvaz. The results of this study showed that art therapy intervention had an effect on increasing the approval, self-concept and happiness of children in the experimental group compared to the control group. They have stated that creating a work of art and enjoying it can both, in addition to growth, bring joy and happiness to the child. Also, the freedom of the art space has added to the joy and happiness of children (24). Based on the results and findings of this study, it is suggested that the study be conducted in a free artistic space for children to have the greatest impact. It can be predicted that this freedom of art space can be observed in the present study by using the presence of more peers. Based on the findings of this study, art therapy is used to create and promote self-confidence and as a result the happiness of children with cancer.  In the study of Dinvari et al. (2015), the effect of painting and music therapy on the hope and happiness of children aged 8-11 in Mahak Hospital has been examined. The sample assessed in this study was 40 children with cancer aged 8-11 in Mahak Hospital in Tehran who were selected by availability sampling method. Participants were selected randomly and were randomly assigned to 4 groups including 3 experimental groups and a control group. The experimental interference groups received painting, music therapy alone, and painting with music therapy, and the control group did not receive any interferences. All 4 groups were examined in two time stages of pre-test and post-test by Snyder Children Oxford Happiness and Hope Scale. The results of analysis of covariance have shown that psychological interference (painting, music, integrative) has a great effect on happiness among children with cancer, so the effect of music therapy, painting therapy and music-painting therapy is significant with 0.99 percent confidence. Also, music-painting interference had an effect on hope in children with cancer, but music and painting interference alone had an effect on hope in children with cancer (29). The findings of this study has been used to benefit from the art of painting to increase happiness in children with cancer in the age range of 7 to 11.  **Results of studies in other parts of the world:**  Increasing happiness by using play therapy in the interference group compared to the control group has also been shown in the study of Grip et al. (2003) who worked on 11 children aged 3-5 with leukemia that are hospitalized. Participants with leukemia (11 patients) were from the external oncology clinic of the urban children's hospital. The children in control group (11) from a day care center have participated. The study assessed children's experiences of stress, social and cognitive play behaviors, and daily moods. It has been shown that. children with leukemia compared to children in control group have significantly increased happiness after interference in a playful behavior. Pearson correlation showed a significant relationship between happiness and play for children with leukemia (30). According to this study, it can be concluded that play therapy is now seen necessary in various forms for children under treatment, especially children with special conditions (with cancer). This study has showed that children with leukemia were able to increase their happiness by play therapy. As it is expected, our study includes children with leukemia in a larger number of testers, so it is predicted that in this study we will be able to gain positive results in the use of art and games to increase children's happiness. In the current study, the role and function of play therapy (painting as one of the main and effective branches of play therapy) in increasing the feeling of happiness and joy of children with cancer and under chemotherapy has been used.  Norris et al. (2012) examined the relationship between artistic activities and positive feeling-happiness in preschool children (3, 4, and 5 years old). A doll is given to each child and they are told that the doll is sad and the children try to make the doll happy. To do this, the children drew a picture of an activity that makes the doll (and actually themselves) happy. In fact, they have been drawing their action to make the doll happy. In this study, art (painting) has shown to have a positive effect on increasing positive emotions (33). In the current study, an attempt was made to use drawing by healthy children to make children with cancer happy.  Finally, according to the positive and remarkable results of research that is done in and outside the country, in the current study we took advantage of all foreign and domestic studies of the role of peers in inducing positive feelings and happiness in children as well as painting tools as one of the most effective branches of art in children, and based on approximate number of sample size and age range, and finally the assessment of level of happiness of children with cancer in relation to artistic interferences such as painting. It should be noted that the innovation of this study is related to a comprehensive study of all the above mentioned things, that has not been done in all the researches together, and in this study, we tried to assess and summarize the relationship between all the mentioned factors and results.    **3- Research Objectives and Goals:**  **A: Main Objective:** Determining the effect of peer painting on the happiness of school aged children with cancer  **Adjunct objectives of the plan:**  Determining and comparing the level of happiness of children before the painting in intervention and control groups  Determining and comparing the level of happiness of children after painting in intervention and control groups  Comparison of the level of children's happiness before and after painting in intervention and control groups  **7.The practical goals of the plan:**  Considering the fact that art has a special and significant place in children's world, and usually what is created in a child's mind in childhood is with them for many years of life (affects his development), the study of the role of art and especially painting are very important in the process of treating and organizing childish behaviors. At present, there is a need for a correct understanding of the country's nursing community the effective role of art in the treatment of some mental illnesses and disorders, especially in children, and the understanding of most medical professionals of the prominent role of art in eliminating psychological anomalies is one of the issues that this study tries to affect. |
| --- |

| **4- Research Questions and Hypothesis:**  Painting by peers has an effect on the happiness of children with cancer. |
| --- |

| **Method:**  The current study is a clinical trial, to assess the effects of drawing on school aged children with cancer that are under treatment (7 to11). To gather the data, the researcher, after gaining permission of the Ethics Committee of School of Nursing and Midwifery and Rehabilitation of Tehran University of Medical Sciences , and by giving a recommendation and obtaining permission of related authorities for sampling will go to the research units. Research population includes children aged 7 to 11 who come to 17 the medical center hospital and have the needed properties and the criteria to participate in the research. The methodology for sampling is availability sampling method. To gather the information, a test of happiness will be used. the test has 20 questions and it has scoring process. When the child has problem about complete the tool the nurse will ask these questions in a childish language so that the child can understand them, then she marks he answers in the answer sheet.  **The type of the study**  The current study will be a clinical trial. In this study, running an interference program is the independent variable, and the children's happiness is the dependent variable.  **Research population:**  The population of this study is all children (aged 7-11) who come to the medical center hospital.  **Place of research:**  The place of research is, the center of children hospital.  **Research sample:**  All children with cancer who come to above mentioned clinic that have the criteria to participate in the research.  **Criteria for participation:**  The child should:  1. Be at age ranged from 7-11 and have a file in the hospital  2. Not have a physical disability for drawing  The peer children who want to participate in the study should:  1.not have a physical disability for drawing  2. aged 7-11  **Exclusion criteria:**  1.Children who avoid drawing for more than two sessions.  2.The death of a child  **Method of calculating the sample size and its number:**  In order to estimate the required sample size at a significant level of 5% and test power 80% and assuming that the effect of peer painting on the happiness of school-aged children with cancer is points (ten percent of the maximum tool performance) so that this effect is considered statistically significant, after putting numbers in the formula:  :  n=$\frac{\left( Z_{1-\frac{\alpha}{2}}+Z_{1-\beta} \right)^{2}*(\sigma_{1}^{2}+\sigma_{2}^{2})}{d^{2}}$  $z_{0.975}=1.96$  $z_{0.8}=0.84$  =10d  The sample size each group was 30 people.  It should be noted that according to the range of changes, the happiness score is 0 to 87 from the formula $\sigma=\frac{R}{6}$=$\frac{87}{6}$The standard deviation was estimated to be 14. In addition, considering the possibility of sample loss, 10% was added to the size of the above sample. Therefore, the sample size in each group was determined to be N = 33.  **Sampling**  Sampling is done by convenience sampling method with random block assignment that consists of two stages. The first stage is selecting the samples that will be done by convenience sampling and the second stage is using the method of assigning the samples to the groups which will be done by random block method random allocation).  **Data analysis method**  First, descriptive statistics will be described by adjusting the frequency distribution tables and calculating the numerical indexes of the samples. Then, by using independent T-tests and K2, the similarity and homogeneity of the variables in the two groups are assessed. Then, using independent t-test and paired t-test and, if necessary, by using analysis of covariance, objectives and hypothesis test will be analyzed. Data analysis will be performed using SPSS software version 16.  **Intervention:**  At first, the researcher, by obtaining permission of the school authorities and in coordination with the relevant teachers, in a meeting with the parents of the children and explaining the objectives and process of the current study, invite them cooperate and complete the form of informed consent. Then by coordinating with the relevant teacher 33 students are selected to perform drawing by covenience sampling method and with a determined schedule (weekly) for 5 sessions and by placing drawing tools (A4 paper, colored pencils, colored markers and crayons in different colors and in sufficient numbers), they are asked to draw for children with cancer who are receiving treatment in the hospital about any subject they want (free and optional subject). Children are justified to use all the tools and in different colors that they want. If the child wants to draw more than one drawing, we will give them an extra A4 paper. The researcher doesn't limit the time of drawing and the child presents their drawing to the researcher whenever they want. It is estimated that the child finishes their painting in about 20 minutes, otherwise we will give them more time and at the end of the session their drawings will be collected. Then the drawings will be taken to the hospital by the researcher every week. In the hospital, after coordination with the hospital director and the officials of the nursing office and the oncology department the work will begin. Samples of the interference and control groups (33 people in each group) will be sampled by convenience method will be assigned to in each group by using random block method (random allocation). Then, the consent form for participating in the research is taken from their parents. Then the happiness questionnaire is also completed before the intervention by explaining the goals and process of the research. After the initial completion of the happiness questionnaire, the researcher asks the children in the intervention group to draw for their healthy peers who are studying at school freely and about any subject that they want. The number of times, duration and conditions of painting in the hospital are the same as in school. The only difference is in the painting place that children draw in the classroom and children with cancer draw in the place where they are hospitalized. Then the drawings of sick children will be given to school children each week and they are asked to draw the next drawing for sick children. And this cycle will continue for 5 weeks. At the end of fifth week and after the complete intervention, the happiness questionnaire in hospitalized children will be completed again.  **Measuring instruments:**  Data collection instruments in this study include:  1- Demographic information questionnaire  2- Children's happiness form  Demographic information questionnaire includes: age, sex, birth rank, parents 'education, parents' job, child's education, weight, age of onset of cancer, duration of cancer, underlying diseases, type of insurance and the habit of painting .  Child happiness questionnaire (Roger Morgan): The Children's Happiness Scale has 20 items and measures the level of child happiness that children or adolescents may determine about themselves. This scale was collected and edited by Dr. Roger Morgan (Director of Child Rights in the UK) and his colleagues from 2001 to March 2014 by reviewing, exploring and reporting the views and perspectives of children under treatment and receive support from the social welfare department and children who are outside t home in boarding schools, educational colleges and other childcare centers aged up to 13-14.  Scoring method of children's happiness questionnaire: This test consists of 20 graded phrases. The highest possible score (happiest) is 4.25. The average score is 2.88 (what researchers call the middle). The lowest possible score is 1.68 (if none of the questionnaires are checked at all). |
| --- |

**6- Variables Table:**

I

|  | **Variable** | **Definition** | **Qualitative** | | **Quantitative** | | **Variable** | | **Measurement**  **Method** | **Scale** |
| --- | --- | --- | --- | --- | --- | --- | --- | --- | --- | --- |
|  |  |  | **Ordinal** | **Nominal** | **Discrete** | **Continuous** | **Independent** | **Dependent** |  |  |
| 1 | happiness | Happiness is an emotional state characterized by feelings of joy, satisfaction, contentment, and fulfillment. While happiness has many different definitions, it is often described as involving positive emotions and life satisfaction. |  |  |  |  |  |  |  |  |
| 2 | Painting/drawing | **Drawing**, the art or technique of producing images on a surface, usually [paper](https://www.britannica.com/technology/paper), by means of marks, usually of [ink](https://www.britannica.com/topic/ink-writing-medium), [graphite](https://www.britannica.com/science/graphite-carbon), chalk, [charcoal](https://www.britannica.com/science/charcoal), or [crayon](https://www.britannica.com/art/crayon). |  |  |  |  |  |  |  |  |
| 3 |  |  |  |  |  |  |  |  |  |  |
| 4 |  |  |  |  |  |  |  |  |  |  |
| 5 |  |  |  |  |  |  |  |  |  |  |
| 6 |  |  |  |  |  |  |  |  |  |  |
| 7 |  |  |  |  |  |  |  |  |  |  |
| 8 |  |  |  |  |  |  |  |  |  |  |
| 9 |  |  |  |  |  |  |  |  |  |  |
| 10 |  |  |  |  |  |  |  |  |  |  |
| 11 |  |  |  |  |  |  |  |  |  |  |
| 12 |  |  |  |  |  |  |  |  |  |  |

**7- Estimated total time to complete the research (in months):**

**8- Research Timeline Table:**

Prepare a list of the activities planned for the research proposed. Mark with X the appropriate cells to reflect the time (each cell represents one month) and duration of each activity.

An example of activities is provided in the first three rows.

|  | **Activities** | **Duration of the activities in months** | | | | | | | | | | | | | | | | | | | | | | | | | | | | | |
| --- | --- | --- | --- | --- | --- | --- | --- | --- | --- | --- | --- | --- | --- | --- | --- | --- | --- | --- | --- | --- | --- | --- | --- | --- | --- | --- | --- | --- | --- | --- | --- |
|  |  | 1 | 2 | 3 | 4 | 5 | 6 | 7 | 8 | 9 | 10 | 11 | 12 | 13 | 14 | 15 | 16 | 17 | 18 | 19 | 20 | 21 | 22 | 23 | 24 |  |  |  |  |  |  |
| 1 | Proposal preparation |  |  |  |  |  |  |  |  |  |  |  |  |  |  |  |  |  |  |  |  |  |  |  |  |  |  |  |  |  |  |
| 2 | Confirmation from the ethics committee |  |  |  |  |  |  |  |  |  |  |  |  |  |  |  |  |  |  |  |  |  |  |  |  |  |  |  |  |  |  |
| 3 | Data gathering |  |  |  |  |  |  |  |  |  |  |  |  |  |  |  |  |  |  |  |  |  |  |  |  |  |  |  |  |  |  |
| 4 | Presentation of report |  |  |  |  |  |  |  |  |  |  |  |  |  |  |  |  |  |  |  |  |  |  |  |  |  |  |  |  |  |  |
| 5 | Writing and publishing article |  |  |  |  |  |  |  |  |  |  |  |  |  |  |  |  |  |  |  |  |  |  |  |  |  |  |  |  |  |  |
| 6 | Presentation of final of research |  |  |  |  |  |  |  |  |  |  |  |  |  |  |  |  |  |  |  |  |  |  |  |  |  |  |  |  |  |  |
| 7 | Total |  |  |  |  |  |  |  |  |  |  |  |  |  |  |  |  |  |  |  |  |  |  |  |  |  |  |  |  |  |  |
| 8 |  |  |  |  |  |  |  |  |  |  |  |  |  |  |  |  |  |  |  |  |  |  |  |  |  |  |  |  |  |  |  |
| 9 |  |  |  |  |  |  |  |  |  |  |  |  |  |  |  |  |  |  |  |  |  |  |  |  |  |  |  |  |  |  |  |
| 10 |  |  |  |  |  |  |  |  |  |  |  |  |  |  |  |  |  |  |  |  |  |  |  |  |  |  |  |  |  |  |  |
| 11 |  |  |  |  |  |  |  |  |  |  |  |  |  |  |  |  |  |  |  |  |  |  |  |  |  |  |  |  |  |  |  |
| 12 |  |  |  |  |  |  |  |  |  |  |  |  |  |  |  |  |  |  |  |  |  |  |  |  |  |  |  |  |  |  |  |

| **9- Ethics: (Attach Ethical Consent Form if applies)**  .  1- Obtaining permission from Ethics Committee of School of Nursing and Midwifery and Rehabilitation of Tehran University of Medical Sciences  2- Submitting recommendation and obtaining a license from the hospitals' management to conduct research  3- Submitting recommendation to the respectable officials of the departments and units under study  4- Explaining the objectives and nature of research for all research units and removing their ambiguities  5- Obtaining written or oral informed consent from the research units and their parents to participate in the study  6- The freedom of the research units to participate in the study or to leave it  7- Assuring the research units that their non-participation or exclusion from the study will not affect their hospitalization process for cancer treatment in the hospital.  8- Assuring the research units and their parents about the confidentiality of the obtained information  9- Considering honesty in sampling, collecting and analyzing data |
| --- |

| **10- Safety Considerations:**  **This research has not any harm for participants** |
| --- |

| **11 - Limitations:**  There is nothing to be mentioned yet.  **Operational Definitions of Terms**  Happiness is an emotional state characterized by feelings of joy, satisfaction, contentment, and fulfillment. While happiness has many different definitions, it is often described as involving positive emotions and life satisfaction.  Drawing, the art or technique of producing images on a surface, usually paper, by means of marks, usually of ink, graphite, chalk, charcoal, or crayon.  **List of abbreviations**  **N/A** |
| --- |

| **12 - References:**  1. Van Rompay KK, Madhivanan P, Rafiq M, Krupp K, Chakrapani V, Selvam D. Empowering the people: Development of an HIV peer education model for low literacy rural communities in India. Human Resources for Health. 2008;6(1):1.  2. Redig AJ, McAllister SS. Breast cancer as a systemic disease: a view of metastasis: a view of metastasis. Journal of internal medicine. 2013;274(2):113-26.  3. Jafroodi M, Y. G. Epidemiologic evaluation of pediatric malignancies in 17 Shahrivar Hospital. Journal of Guilan University of Medecine Science  200; (68):14-21.  4. Mirzaie M YF, Navidi Z. . Survey personal and disease characteristics of children with cancer hospitalized in 17 shahrivar hospital, Rasht. Journal of Guilan Faculty of Medicine. 2010;19(61): 32-6.  5. Zareapour A, Falahi Khoshknab M, Kashaninia Z, Biglarian A, R. B. Effect of group play therapy on depression in children with cancer. . Scientific Journal of Kurdistan University of Medical Sciences. 2009; 14(3):64-72.  6. Mousavi SM, Pourfeizi A, Dastgiri S. Childhood cancer in Iran. Journal of pediatric hematology/oncology. 2010;32(5):376-82.  7. Hockenberry MJ, Wilson D, Wong DL. Wong's Essentials of Pediatric Nursing9: Wong's Essentials of Pediatric Nursing: Elsevier Health Sciences; 2012.  8. علیلو م, آباد هن, صفت فم. اثربخشی بازی درمانی براساس رویکرد لوی در کاهش اضطراب کودکان سرطانی. مجله پرستاری و مامایی جامع نگر. 2015;25(1):54-62.  9. Marlow DR, B. Text book of pediatric, 2010 nPWBSC.  10. Varni JW, Katz ER, Colegrove Jr R, Dolgin M. Perceived physical appearance and adjustment of children with newly diagnosed cancer: A path analytic model. Journal of Behavioral Medicine. 1995;18(3):261-78.  11. Larcombe I, Walker J, Charlton A, Meller S, Jones PM, Mott M. Impact of childhood cancer on return to normal schooling. BMJ. 1990;301(6744):169-71.  12. Susman EJ, Hollenbeck AR, Nannis ED, Strope BE, Hersh SP, Levine AS, et al. A prospective naturalistic study of the impact of an intensive medical treatment on the social behavior of child and adolescent cancer patients. Journal of Applied Developmental Psychology. 1981;2(1):29-47.  13. Jakobsson S, Ekman T, Ahlberg K, editors. Components that influence assessment and management of cancer-related symptoms: an interdisciplinary perspective. Oncology nursing forum; 2008.  14. Compas BE, Desjardins L, Vannatta K, Young-Saleme T, Rodriguez EM, Dunn M, et al. Children and adolescents coping with cancer: self-and parent reports of coping and anxiety/depression. Health Psychology. 2014;33(8):853.  15. Pinquart M, Teubert D. Academic, physical, and social functioning of children and adolescents with chronic physical illness: a meta-analysis. Journal of pediatric psychology. 2011:jsr106.  16. Alderfer MA, Hodges JA. Supporting siblings of children with cancer: A need for family–school partnerships. School mental health. 2010;2(2):72-81.  17. Michel G, Rebholz CE, Nicolas X, Bergstraesser E, Kuehni CE. Psychological distress in adult survivors of childhood cancer: the Swiss Childhood Cancer Survivor study. Journal of Clinical Oncology. 2010;28(10):1740-8.  18. Durualp E, Altay N. A Comparison of Emotional Indicators and Depressive Symptom Levels of School-Age Children With and Without Cancer. Journal of Pediatric Oncology Nursing. 2012;29(4):232-9.  19. Holder MD, Coleman B, Singh K. Temperament and happiness in children in India. Journal of Happiness Studies. 2012;13(2):261-74.  20. Gillespie Edwards A. Self Concept in Relationships and Learning, Caring for Childre  from Birth to Three. London: : National Children’s Bureau/PEEP; (2002).  21. Katz C, Hamama L. “Draw me everything that happened to you”: Exploring children's drawings of sexual abuse. Children and Youth Services Review. 2013;35(5):877-82.  22. Rollins JA. Tell me about it: drawing as a communication tool for children with cancer. Journal of Pediatric Oncology Nursing. 2005;22(4):203-21.  23. Councill T. Medical art therapy with children. Handbook of art therapy. 2003:207-19.  24. Nix GA, Ryan RM, Manly JB, Deci EL. Revitalization through self-regulation: The effects of autonomous and controlled motivation on happiness and vitality. Journal of Experimental Social Psychology. 1999;35(3):266-84.  25. Yost E, Ellis GD. Effect of Self Determination Theory-Based Recreation Activity-Staging on Vitality and Affinity Toward Nature Among Youth in a Residential Treatment Program. Residential Treatment for Children & Youth. 2008;23(1-2):5-26.  26. Vygotsky LS. Mind in society: The development of higher mental process. Cambridge, MA: Harvard University Press; 1978.  27. Tharp RG, Gallimore R. Rousing minds to life: Teaching, learning, and schooling in social context: Cambridge University Press; 1991.  28. Taghdisi M, NOORI SM, MERGHATI KE, Hoseini F, ASGHARNEJAD FA. Impact peer education approach on knowledge and practice about Mental Health of Adolescent Girls. 2012.  29. MOTEVASELIAN M, NASIRIANI K. Impact of Near-peer teaching on Learning Dressing Skill among Nursing Students. 2014.  30. Borzou R, Bayat Z, Salvati M, Homayounfar S. A comparison of Individual and Peer Educational Methods on Quality of life in patients with heart failure. Iranian Journal of Medical Education. 2014;14(9):767-76.  31. Thomas AM, Peterson L, Goldstein D. Problem solving and diabetes regiman adherence by children and adolescents with IDDM in social pressure situations: A reflection of normal development. Journal of Pediatric Psychology. 1997;22(4):541-61.  32. Shirazi M, Anoosheh M, Rajab A. The effect of self care program education by group discussion method on self concept in diabetic adolescent girls reffered to Iranian Diabetes Society. 2011.  33. Hamre HJ, Witt CM, Glockmann A, Ziegler R, Willich SN, Kiene H. Anthroposophic art therapy in chronic disease: a four-year prospective cohort study. Explore: The Journal of Science and Healing. 2007;3(4):365-71.  34. Jones EM, Landreth G. The efficacy of intensive individual play therapy for chronically ill children. International Journal of play therapy. 2002;11(1):117.  35. MAMIYANLOU H, ELHANI F, GHOFRANIPOUR F. Evaluation of the Effects of a Play Program on the Anxiety of Hospitalized School-Age Children in Tehran Medical Center of Children in 1999-2000. 2001.  36. دینور م, شمس اسفند آبادی ح, جلالی م. بررسی تاثیر نقاشی و موسیقی درمانی در شادکامی و امید کودکان سرطانی. تهران: دانشگاه بین‌المللی امام خمینی (ره); 1394.  37. نیکنامی, یعقوبی, یاسمن, پور ش, عطرکارروشن, نیا م. ‏ بررسی تأثیر بازی برمیزان اضطراب کودکان سن دبستان بستری در مرکز آموزشی-‏ درمانی کودکان گیلان. مجله پرستاری و مامایی جامع نگر. 2005;15(1):65-71.  38. Larigani B AF, Mohagery Tehrani MR, Tabatabaee A. . frequency Diabetics type 2 in Iran in year of 1380. Journal Diabetes & Lipid Iran ; . 1384; 4(3):75-82.  39. نادری ف, زينب اا. تاثير هنر درماني بر خودپنداره تاييدجويي و شادکامي کودکان مراجعه کننده به مراکز مشاوره و درمان شهرستان اهواز.  40. افسانه زپ, مسعود فخ, زهرا كن, اكبر ب, روناك ب. بررسي تاثير بازي درماني گروهي بر ميزان افسردگي كودكان مبتلا به سرطان.  41. Bolton P, Bass J, Betancourt T, Speelman L, Onyango G, Clougherty KF, et al. Interventions for depression symptoms among adolescent survivors of war and displacement in northern Uganda: a randomized controlled trial. Jama. 2007;298(5):519-27.  42. Noll RB. Evaluation of the school competency assessment scale: A critique. Journal of Pediatric Oncology Nursing. 2003;20(2):65-70.  43. López-Pérez B, Sánchez J, Gummerum M. Children’s and Adolescents’ Conceptions of Happiness. Journal of Happiness Studies. 2016;17(6):2431-55.  44. نعيمه ب, مهرنوش ن. اثر تکنيک هاي بازي درماني در کاهش اضطراب و افزايش احساسات مثبت و سطح سازگاري عمومي در کودکان 9-12 ساله مبتلا به سرطان خون.  45. Jangi S, Shirabadi A, Ansarhosein S, Ghoradel JA. Effecacy of painting therapy in reducing aggression in children with conduct disorder. Journal of Kermanshah University of Medical Sciences (J Kermanshah Univ Med Sci). 2014;18(8):443-51.  46. جنگی, شیرابادی, جانی, ستاره, پوراسمعلی. اثربخشی هنر‌درمانی بر پایه نقاشی‌ درمانی در کاهش اضطراب کودکان دچار لکنت زبان. مجله علمی پزوهشی دانشگاه علوم پزشکی ایلام. 2015;23(2):53-60.  47. فرامرزی, سالار, مرادی. تأثیر هنردرمانی با رویکرد نقاشی بر کاهش ناامیدی و تنهایی کودکان ناشنوای پسر. شنوایی شناسی-دانشگاه علوم پزشکی تهران. 2015;23(6):25-31.  48. پیریایی آ, پیریایی آ. تأثیر طراحی داخلی در بیمارستان کودکان سرطانی با رویکرد مدیریت استرس و تسریع بهبودی آن‌ها  بیمارستان فوق تخصصی کودکان محک تهران.  49. شیخذکریایی ن, کریدی گ, اردلان م. تاثير نقاشي بر اضطراب کودکان سرطاني بستري در بيمارستان.  50. ATTARI B, SHAFI AA, SALIMI H. The Effectiveness of Teaching Painting on Decreasing the Amount of Anxiety in Elementary School Boys. 2012.  51. Khodabakhshi Koolaee A, Vazifehdar R, Bahari F. Impact of painting therapy on aggression and anxiety of children with cancer. Caspian Journal of Pediatrics. 2016;2(2):135-41.  52. جهانگير ك, مصطفي ع, علي زي, كامران خ. اثربخشي نقاشي درماني در کاهش رفتارهاي پرخاشگرانه‌ دانش ‌آموزان دختر دچار نارساخواني.  53. نریمانی, بشرپور, سجاد, صومعه ع, سجاد. مقایسه عزت نفس و شادکامی در دانش آموزان ناشنوای مدارس تلفیقی و استثنایی. 2014.  54. Khadar MG, Babapour J, Sabourimoghaddam H. The effect of art therapy based on painting therapy in reducing symptoms of oppositional defiant disorder (ODD) in elementary School Boys. Procedia-Social and Behavioral Sciences. 2013;84:1872-8.  55. Khadar MG, Babapour J, Sabourimoghaddam H. The Effect of Art Therapy based on Painting Therapy in Reducing Symptoms of Separation Anxiety Disorder (SAD) in Elementary School Boys. Procedia - Social and Behavioral Sciences. 2013;84:1697-703.  56. خادر غ, باباپور, مقدم ص. اثربخشی نقاشی درمانی در کاهش نشانه های اختلال افسردگی کودکان دبستانی. پژوهش های روان‌شناسی بالینی و مشاوره. 2015;4(2):19-32.  57. Mousavi M, Sohrabi N. Effects of art therapy on anger and self-esteem in aggressive children. Procedia-Social and Behavioral Sciences. 2014;113:111-7.  58. Nezamipour EAA, Atefe%A Etemadinia, Mahin%A Ezadinia, Nasrin. The Efficacy of Drawing-Therapy on Reducing Aggressive Behavior of Hard Hearing Student. Journal of Exceptional Education. 2015;3(131):31-8.  59. Gariépy N, Howe N. The therapeutic power of play: examining the play of young children with leukaemia. Child: care, health and development. 2003;29(6):523-37.  60. Favara‐Scacco C, Smirne G, Schilirò G, Di Cataldo A. Art therapy as support for children with leukemia during painful procedures. Medical and pediatric oncology. 2001;36(4):474-80.  61. Barrera ME, Rykov MH, Doyle SL. The effects of interactive music therapy on hospitalized children with cancer: a pilot study. Psycho-oncology. 2002;11(5):379-88.  62. Sadruddin MM, Hameed-ur-Rehman M. Understanding the perceptions of children battling cancer about self and others through drawing. South Asian journal of cancer. 2013;2(3):113.  63. McCaffrey CN. Major stressors and their effects on the well-being of children with cancer. Journal of Pediatric Nursing. 2006;21(1):59-66.  64. de Mello Sabino MB, de Amorim Almeida F. Therapeutic play as a pain relief strategy for children with cancer. Einstein. 2006.  65. Holder MD, Coleman B. The contribution of temperament, popularity, and physical appearance to children’s happiness. Journal of Happiness Studies. 2008;9(2):279-302.  66. Holder MD, Coleman B, Wallace JM. Spirituality, religiousness, and happiness in children aged 8–12 years. Journal of Happiness Studies. 2010;11:131-50.  67. Norris A. Children and Art: Exploring the Correlation between Art Activities and Positive Emotion/Happiness in Preschoolers. 2012.  68. Puetz TW, Morley CA, Herring MP. Effects of creative arts therapies on psychological symptoms and quality of life in patients with cancer. JAMA internal medicine. 2013;173(11):960-9.  69. Tsai Y-L, Tsai S-C, Yen S-H, Huang K-L, Mu P-F, Liou H-C, et al. Efficacy of therapeutic play for pediatric brain tumor patients during external beam radiotherapy. Child's Nervous System. 2013;29(7):1123-9.  70. Giacomoni CH, Souza LKd, Hutz CS. O conceito de felicidade em crianças. Psico USF. 2014;19(1):143-53.  71. Brown J. Group medical play for reducing stress and improving mood in children going to visit the pediatrician: The University of Alabama TUSCALOOSA; 2012.  72. do Vale Pinheiro I, da Costa AG, Rodrigues DCB, de Paula Oliveira N, Malheiro A, Ramos JL. Hospital psychological assessment with the drawing of the human figure: A contribution to the care to oncologic children and teenagers. Psychology. 2015;6(04):484.  73. به‌پژوه, نوری. تأثیر نقاشی درمانی در کاهش رفتارهای پرخاشگرانه دانش آموزان عقب مانده ذهنی. روانشناسی و علوم تربیتی (دانشگاه تهران). 2002;2(32):155-70.  74. Dr Roger Morgan OBE ,The children's happiness scale ,March 2014  .75 Cartagena RG, Veugelers PJ, Kipp W, Magigav K, Laing LM, Effectiveness of an HIV prevention program for secondary school students in Mongolia . Journal of Adolescent Health . 2006; 39(6): 925. e9-e16  .76-اصفهانی،زهرا-رضازاده ،مجید-سیگاری،سپیده-فرهودی،بهنام-احترامی،مهرداد-شیبانی،شهناز و دیگران(1389)آموزش پیشگیری از ایدز برای افراد در معرض خطر.تهران،ایران:دانشگاه علوم پزشکی بقیه ا...(عج)،مرکز تحقیقات علوم رفتاری. |
| --- |

**13- Budget Details: (in Rials)**

| **List of Expenses** | | | | | | **Cost (amount in Rials)** |
| --- | --- | --- | --- | --- | --- | --- |
| Research Personnel and Partners’ Compensation | **Name** | | **Hours required** | | |  |
|  | 1. Somayee Palvan | |  | | | 20000000 |
|  | 1. Dr. A. Sadat Hosseini | |  | | | 5000000 |
|  | 1. Khadije Zaree | |  | | | 3000000 |
|  |  | |  | | |  |
|  |  | |  | | |  |
|  | 6- | |  | | |  |
|  | 7- | |  | | |  |
|  | 8- | |  | | |  |
| **Subtotal:** | | | | | | 28000000 |
| Equipment and instruments  (non expendable) | **Equipment and Model No.** | | **Manufacturer** | | **Quantity** |  |
|  | 1. Cryon/ pencile | | China | | 1 | 1000000 |
|  | 1. Paper/pencil | | Germany | | 1 | 5000000 |
|  |  | |  | |  |  |
|  | 4- | |  | |  |  |
|  | 5- | |  | |  |  |
|  | 6- | |  | |  |  |
| **Subtotal:** | | | | | | 6000000 ­­­­­­ |
| Equipment (expendable),  lab animals and Materials | **Items** | | **Manufacturer or Provider** | | **Quantity** |  |
|  | 1- | |  | |  |  |
|  | 2- | |  | |  |  |
|  | 3- | |  | |  |  |
|  | 4- | |  | |  |  |
|  | 5- | |  | |  |  |
|  | 6- | |  | |  |  |
|  | 7- | |  | |  |  |
|  | 8- | |  | |  |  |
|  | 9- | |  | |  |  |
|  | 10- | |  | |  |  |
|  | 11- | |  | |  |  |
|  | 12- | |  | |  |  |
|  | 13- | |  | |  |  |
|  | 14- | |  | |  |  |
|  | 15- | |  | |  |  |
| Lab Tests and Services  (specify) | **Laboratory Name** | | **Number of Tests** | | |  |
|  |  | |  | | |  |
|  |  | |  | | |  |
|  |  | |  | | |  |
|  |  | |  | | |  |
|  |  | |  | | |  |
|  |  | |  | | |  |
|  |  | |  | | |  |
|  |  | |  | | |  |
|  |  | |  | | |  |
|  |  | |  | | |  |
| Travel | Destinations | Transport Mode | Number of People Travelling | Number of trips | |  |
|  |  |  |  |  | |  |
|  |  |  |  |  | |  |
|  |  |  |  |  | |  |
| Books, Copy and Print | Specify: Paper, photocopies, printing of letters of permission | | | | | 2000000 |
| Communication (phone, web etc) | Specify: phone calls, official communique with departmental heads | | | | | 2000000 |
| Other Expenditures |  | | | | | 0 |
| **Grand Total:** | | | | | |  |

**Important Note: For the year 2014**

• The maximum funding which may be assigned to **M.Sc.** theses is **25,000,000 Rials.**

• The maximum funding which may be assigned to **Ph.D.** theses is **75,000,000 Rials.**

• The maximum funding which may be assigned to **Undergraduate** theses is **15,000,000 Rials.**

**14- Are you going to receive any financial assistance/budget from other sources for this research? (if yes, specify where and how much)**

**Project Manager(s) Affirmation:**

**Full Name:**

**Signature**

**Date**

**1-**

**2-**

**3-**
