# Supplementary material for: The effect of exchanging drawings with peers on the happiness of children with cancer, aged 7–11 years: A clinical trial
Source: PLoS One. 2021 Oct 15;16(10):e0257867. doi: 10.1371/journal.pone.0257867 (PMC8519419; doi:10.1371/journal.pone.0257867)
Supplement: Attachment — Submitted filename: Response to reviewer.23-8.docx [file pone.0257867.s006.docx]

**Response to reviewer**

Dear Editor,

We are grateful for your meticulous review of this manuscript, and your suggestions, which have been very helpful in improving its quality. We also thank the reviewers for their careful reading and valuable suggestions. All the comments we received on this study have been addressed and replied separately. We hope that these changes to the manuscript will facilitate the decision to publish this study in your journal.

| reviewer | comments | Correction and responses | Page and place |
| --- | --- | --- | --- |
| editor | . Please ensure that your manuscript meets PLOS ONE's style requirements, including those for file naming. | corrected | All file |
|  | Please include additional information regarding the survey or questionnaire used in the study and ensure that you have provided sufficient details that others could replicate the analyses. For instance, if you developed a questionnaire as part of this study and it is not under a copyright more restrictive than CC-BY, please include a copy, in both the original language and English, as Supporting Information.  If the original language is written in non-Latin characters, for example Amharic, Chinese, or Korean, please use a file format that ensures these characters are visible. If the questionnaire can not be published CC-BY, please include a reference | They are attached and explained in the text |  |
|  | Thank you for submitting your clinical trial to PLOS ONE and for providing the name of the registry and the registration number. The information in the registry entry suggests that your trial was registered after patient recruitment began. PLOS ONE strongly encourages authors to register all trials before recruiting the first participant in a study.  As per the journal’s editorial policy, please include in the Methods section of your paper: 1) your reasons for your delay in registering this study (after enrolment of participants started); 2) confirmation that all related trials are registered by stating: “The authors confirm that all ongoing and related trials for this drug/intervention are registered”.  Please also ensure you report the date at which the ethics committee approved the study as well as the complete date range for patient recruitment and follow-up in the Methods section of your manuscript. | Corrected and added in methods | Page9 |
|  | Thank you for stating the following in the Acknowledgments Section of your manuscript:  "This study was part of a M.S. thesis supported by Tehran University of Medical Sciences grant NO 9311700002 and IRCT registration number: IRCT20150928024239N4" | Thanks  Added in cover letter about funding |  |
|  | We note that you have provided funding information that is not currently declared in your Funding Statement. However, funding information should not appear in the Acknowledgments section or other areas of your manuscript. We will only publish funding information present in the Funding Statement section of the online submission form.  Please remove any funding-related text from the manuscript and let us know how you would like to update your Funding Statement. Currently, your Funding Statement reads as follows:  "no"  Please include your amended statements within your cover letter; we will change the online submission form on your behalf. |  |  |
|  | Your ethics statement should only appear in the Methods section of your manuscript. If your ethics statement is written in any section besides the Methods, please move it to the Methods section and delete it from any other section. Please ensure that your ethics statement is included in your manuscript, as the ethics statement entered into the online submission form will not be published alongside your manuscript. | Corrected | Page 11 |
|  | Please include captions for your Supporting Information files at the end of your manuscript, and update any in-text citations to match accordingly | Corrected |  |
| Reviewer 1 | Page 6, information on the sample size calculation to be provided i.e. alpha, beta, 1 or 2 tailed test, outcome measure, attrition rate consideration etc | Corrected | Page7 |
|  | Page 6, information on who prepared the randomization block (sequence generation/allocation, concealment) and assigned to the groups to be clearly stated. | Corrected | Page7 |
|  | Page 6, the word control group or peer group to be used systematically. The write-up on the subjects selection to be placed on top first before the randomization process. | Corrected | Page 7 &8 |
|  | Page 7, the sentence 'The exclude their data from analysis for both groups consisted of absence from more than two sessions of painting or death of the child.' to be revised. | Corrected | Page 8 |
|  | Page 7, the language version of the Children's Happiness Scale used in the study to be stated. If it is Iranian version the validation information to be provided/cited. | Corrected | Page8 &9 and supplement in the article the original version of tool |
|  | Page 7, information on blinding (providers, subjects, person assessing the outcome) to be stated. | Corrected | Page12 |
|  | Page 7, is the lowest score for Children's Happiness Scale 1.67 or 1.68? | Corrected | Page8 |
|  | Page 7, for the reliability test of the scale, the exact name of the correlation test to be stated. The range of the correlation values to be provided. | Corrected | Page 9 |
|  | Page 10, the sentence ‘In this cases were sent peer’s painting through email’ & ‘Researcher ask their to draw a painting’ to be revised. | Corrected | Page12 |
|  | Page 10, a sub-title for the statistical analysis to be provided. | Corrected | Page 12 |
|  | Page 10, proper citation of SPSS and publisher name to be provided. The use of the independent T-test, the Chi-square test, Fisher's exact test and the paired T-test and its purpose to be clearly stated. | Corrected | Page 12 |
|  | The acceptance level of significance to be stated. | Corrected | Page 12 |
|  | Page 10, study limitation to be placed in the discussion section. | Corrected | Page 17 |
|  | Page 12, Kolmogorov-Smirnov test to be stated in the statistical analysis section. | Corrected | Page 12 |
|  | Page 12, the results with no significant differences in the children's happiness before and after painting in the control group to be stated in the text. | Corrected | Page 13 |
|  | Page 12, the write-up of the results section is too short. | Yes of course but I explain all criteria in tables |  |
|  | Page 19 N for each group to be stated for Table 1 and 2. | Corrected | In the file of table was corrected |
|  | Page 19 Table 1, the alignment of the table and words to be improved. Decimal point for p value to be provided and standardized. Chi-square test, Fisher’s Exact Test to be denoted in table/table footnote. Symbol % for individual figure to be omitted since it has been highlighted in the group name. Word P value to be stated in the last column. | Corrected | In the file of table was corrected |
|  | Page 19 Table 2, t-statistic value, df to be placed in another column/row from the p value. Alignment of the table and words to be improved. | corrected | In the file of table was corrected |
|  | It would be good to display/describe the number of subjects who were not happy/happy before intervention and after intervention other than describing the mean score of the scale. | The scale did not have a cutoff point to determine happy/unhappy children and only reported the mean level of happiness. Therefore, the research team could not divide the children into happy and unhappy groups. |  |
|  | Page 12 & 19, the focus to be more on within group comparison rather than comparison between the groups at each time period since the two groups of subjects are different. Mean difference (pre-post) from each group could be used for between group comparison. Effect size i.e. Cohen’s d and 95% confidence interval to be provided. | corrected | Do in the file of table 2 |
|  | Page 19, more baseline characteristics of the study subjects to be provided such as types/ severity of cancer (stages), type of medications, severity of pain, surroundings/environment etc | We collected and recorded the data that we found in the patients’ files such as the type of cancer (they were all ALL) and that all underwent chemotherapy, but no other information was recorded in their files and unfortunately, we could not report other data. | Table 1 in the file of table |
|  | Some references did not conform to the journal format. Bracket symbol ( ) for reference in the text to be replaced with [ ]. | Corrected |  |
|  | For the CONSORT Flow diagram, group name, assessment period, outcome measure(s) to be included. | Corrected | In the file of consort was attached |
|  | The write-up of the manuscript can be further improved in terms of grammar and presentations | Did it | In the all section of manuscript |
| Reviewer 2 |  |  |  |
|  | Very creative, interesting, and well done study. Congratulations! Sorry that you didn't get the results you were hoping for, but you have done an excellent job in analyzing the limitations and how such a study could be done differently to possibly achieve more positive results. | Thanks for attention I hope in future research we reduce limitation and get positive results |  |
|  | . The term "painting" is used throughout. However, the list of materials used does not include paint (or paint brushes), which defines painting for many people. From the materials used, I believe the term "drawing" is more accurate. Thus, the title would be "The Effect of Exchanging Drawings...." and other replacements for "painting" and "paintings" made throughout the manuscript. | Corrected | In the manuscript |
|  | 2. Page 10, Line 10, "an expert in art-therapy." In many countries the term art therapy means something very specific: Art therapy is a regulated mental health profession that requires a master's or doctoral level education in Art Therapy, clinical hours, supervision. and licensure. There is a credentialing and certification process. As I did not see art therapy credentials after any of the authors' names, I'm assuming that to say art therapy could be misleading to an international audience. The phrase "an expert nurse in the therapeutic use of the arts" would be more accurate and help to avoid confusion and criticism from the art therapy community. | Thanks for your valuable points. All the mentioned points are correct and the manuscript was modified accordingly. | Page 12 |
|  | A question: the children were instructed that they could draw whatever they wanted. Were the school children told that the purpose of the drawings were to try to make children in the hospital happy? Please clarify. | No, school children were only told to draw some pictures for children with cancer. The study objectives were explained to children and their parents when we were going to obtain informed consent. |  |
|  | Gender bias: avoid the use of "he" in the general sense, instead use "he/she" or "he and she" or rewrite the sentence in the plural and using "they." | Corrected | Page 8 |
|  | I encourage you to continue to explore and expand on this exciting peer to peer intervention. | Thank you for your kindness and valuable suggestion. |  |
|  | The purpose of this study is to examine the effect of exchanging paintings with peers on the happiness of children ages 7-11 years using a clinical trial. It sounds like an innovative technique to improve the happiness of children; however, the study design could not provide convincing results for health professionals to further investigate if this technique would really work. Therefore, better study design is needed to improve children’s psychological health. | Thanks for your great advice. The researchers noticed limitations during this study, too, which are mentioned in the Limitations section. Since this was the first experience of this kind of study in Iran, it certainly had some shortcomings that we hope will be eliminated in the future studies with improved designs. Nonetheless, we tried to clarify the ambiguities in the methods section during revision. |  |

Best: Sadat Hoseini
