# Supplementary material for: The effect of exchanging drawings with peers on the happiness of children with cancer, aged 7–11 years: A clinical trial
Source: PLoS One. 2021 Oct 15;16(10):e0257867. doi: 10.1371/journal.pone.0257867 (PMC8519419; doi:10.1371/journal.pone.0257867)
Supplement: Attachment — Submitted filename: Response to reviewer.plos-palvan 28-12.docx [file pone.0257867.s007.docx]

**Response to reviewer**

Dear Editor,

We are grateful for your meticulous review of this manuscript, and your suggestions, which have been very helpful in improving its quality. We also thank the reviewers for their careful reading and valuable suggestions. All the comments we received on this study have been addressed and replied separately. We hope that these changes to the manuscript will facilitate the decision to publish this study in your journal.

| Reviewer 1 | | |
| --- | --- | --- |
| comment | answer | place |
| Table 1, if the expected cells less than 5 more than 20% e.g. 'variable 'interested in drawing'' chi-square test is not suitable. Decimal point for p value to be standardized. At least one decimal point for percentage value, Cohen D to be denoted in table footnote or in statistical analysis section for what comparison. 'No'' to be replaced with n. | Was corrected | Page 23 table 1  And file table |
| Table 2, the row for the paired t-test to be placed in the last row after mean difference. Decimal point for p value, SD, t value to be standardized. (%95Confidence Interval to be written as 95% Confidence Interval. *T-Test to be replaced with *Independent t-test | Was corrected | Page 23 table 2  And file table |
| Ensure all the statistical tests used in the study are stated in the statistical analysis section. | Was added  using the independent t-test and paired t-test for comparison of the happiness scores between the two groups , the Chi-square test for comparison of the demographics, Fisher’s exact test for interest in drawing and paired t-test for comparison of the happiness scores in each group | Page 13 |
| Reviewer2 | | |
| The revision brings more clarity regarding the intervention and other methods. Several instances of "painting" have not been changed to "drawing" e.g., page. 9, line 15, page 10, line 14, page 15, lines 10 and 12, and perhaps others. | All of them was changed  I hope did all of them and have not mistake | whole of paper |
| Gender bias remains, page 11, line 5. Also check for typos. | Was corrected  The drawing intervention sessions were held after the child had received their medications and been visited by the doctors to avoid disturbing the child during the process of drawing. | Page 11 |
| In the discussion session, some examples are given e.g., reference 38) that were facilitated by art therapists, which could account for success because although it may look the same, the process and goals can be different. If art therapy examples are going to be used, the difference needs to be identified. | Was corrected  Although the process and goals of drawing therapy and drawing are not similar, this research showed benefit of the process of drawing for reducing behavior problems. | Page 16 |
| Reviewer3 | | |
| Thank you for addressing the reviewers comments in your paper. The revised version has improved a greatly deal comparing with the previous version. | Thanks for attention |  |
| However, much improvement, especially in academic writing is needed for the future revision. I attached the detailed comments for your reference. | Was reedited  Unfortunately I did not received attach file. I send some email but I cannot get it. | Whole of paper |
