# Supplementary material for: The effect of exchanging drawings with peers on the happiness of children with cancer, aged 7–11 years: A clinical trial
Source: PLoS One. 2021 Oct 15;16(10):e0257867. doi: 10.1371/journal.pone.0257867 (PMC8519419; doi:10.1371/journal.pone.0257867)
Supplement: Attachment — Submitted filename: plos-palvan-revised-12-1.docx [file pone.0257867.s008.docx]

**Response to reviewer**

Dear Editor,

We are grateful for your meticulous review of this manuscript, and your suggestions, which have been very helpful in improving its quality. We also thank the reviewers for their careful reading and valuable suggestions. All the comments we received on this study have been addressed and replied separately. We hope that these changes to the manuscript will facilitate the decision to publish this study in your journal.

| Reviewer1 | | |
| --- | --- | --- |
| comments | answer | place |
| The derived p value whether based on 1 or 2-tailed test to be stated. | Was corrected | In statistical section and table 2 |
| List of references did not conform to the journal format. | All references conform to the journal format and some mistake was corrected and one reference was deleted for duplication write | References section |
| Reviewer2 | | |
| Page 12, line 5, “force” sounds too aggressive; suggest using “encourage.” | Was corrected | Page 5 |
| Page 14, line 8, change uppercase N to lower case n because it represents part of the total sample, N = 66, n =33. | Was corrected | Page 7 |
| Page 17, line 7-8, “brief the children’s teachers and parents on the study objectives…” Were the children told than an objective of the study was to make children with cancer happier? | Base on the consent form of TUMS the research team must be explain purpose and subject of research to participant and they must be sign this form .” I, , have been explained about the ***purpose & objectives*** of this research and I am aware of them. The purpose& objectives are as follows: This Survey is about the Effect of Exchanging Paintings with Peers on the Happiness of Children with Cancer” | The consent form was attached the time of submission.  If it is necessary, I can write on the manuscript this sentence.(in the box of answer). Currently, I write on the manuscript “. A meeting was then arranged to brief the children’s teachers and parents on the study objectives and methods”. |
| Page 19, line 9, typo “ad” to “and” | Was corrected |  |
| Journal Requirements: | | |
| Please review your reference list to ensure that it is complete and correct. If you have cited papers that have been retracted, please include the rationale for doing so in the manuscript text, or remove these references and replace them with relevant current references. Any changes to the reference list should be mentioned in the rebuttal letter that accompanies your revised manuscript. If you need to cite a retracted article, indicate the article’s retracted status in the References list and also include a citation and full reference for the retraction notice. | All references was checked and conform to journal form.  One of the references was deleted for retyping  Some references were corrected and others were updated  All change show with highlighted | References section  1. Jafroodi M. Epidemiologic evaluation of pediatric malignancies in 17 Shahrivar Hospital. Journal of Guilan University of Medecine Science. 2000;68:14-21. Persian.  2. Redig A, McAllister S. Breast cancer as a systemic disease: a view of metastasis: a view of metastasis. Journal of internal medicine. 2013;4(2):113-26.  3. Holder M, Coleman B, Singh K. Temperament and happiness in children in India. Journal of Happiness Studies. 2012;13(2):261-74.  4. Katz C, Hamama L. Draw me everything that happened to you”: Exploring children's drawings of sexual abuse. Children and Youth Services Review. 2013;35(3): 877-82.  5. Mehranfar M, Younesi J, Banihashem A. Effectiveness of mindfulness-based cognitive therapy on reduction of depression and anxiety symptoms in mothers of children with cancer. Iran J Cancer Prev. 2012;5(1):1-9. Epub 2012/01/01. PubMed PMID: 25780532; PubMed Central PMCID: PMCPMC4352519 Persian..  6. Verkooijen HM. [Happiness and cancer]. Ned Tijdschr Geneeskd. 2012;156(51):A5752. Epub 2012/12/20. PubMed PMID: 23249517.. Dutch.  7. Rogers MA, Zaragoza-Lao E. Happiness and Children’s Health: An Investigation of Art, Entertainment, and Recreation. Am J Public Health. 2003;93(2):288-9. doi: 10.2105/ajph.93.2.288.  8. Dickie K, Holder MD, Binfet JT. Happiness in Children. [cited 27 July 2017]. Availiable from: https://www.oxfordbibliographies.com/view/document/obo-9780199791231/obo-9780199791231-0188.xml.  9. Rollins J. Tell me about it: drawing as a communication tool for children with cancer. Journal of Pediatric Oncology Nursing. 2005;22(4): 203-21.  10. Badri M, Al Nuaimi A, Guang Y, Al Sheryani Y, Al Rashedi A. The effects of home and school on children’s happiness: a structural equation model. International Journal of Child Care and Education Policy. 2018;12(1):17. doi: 10.1186/s40723-018-0056-z.  11. Ackerman C. What is Positive Psychology & Why is It Important. [cited 06.12.2020]. Available from: <https://positivepsychology.com/what-is-positive-psychology-definition/>.  12. Bekhet AK, Zauszniewski JA, Nakhla WE. Happiness: theoretical and empirical considerations. Nurs Forum. 2008;43(1):12-23. Epub 2008/02/14. doi: 10.1111/j.1744-6198.2008.00091.x. PubMed PMID: 18269440.  13. Chaves C, Hervas G, García FE, Vazquez C. Building Life Satisfaction Through Well-Being Dimensions: A Longitudinal Study in Children with a Life-Threatening Illness. Journal of Happiness Studies. 2015;17(3):1051-67. doi: 10.1007/s10902-015-9631-y.  14. Hendriks T, Schotanus-Dijkstra M, Hassankhan A, de Jong J, Bohlmeijer E. The Efficacy of Multi-component Positive Psychology Interventions: A Systematic Review and Meta-analysis of Randomized Controlled Trials. Journal of Happiness Studies. 2019;21(1):357-90. doi: 10.1007/s10902-019-00082-1.  15. Kubzansky LD, Kim ES, Salinas J, Huffman JC, Kawachi I. Happiness, health, and mortality. Lancet. 2016;388(10039):27. Epub 2016/07/12. doi: 10.1016/S0140-6736(16)30896-0. PubMed PMID: 27397787.  16. Mahon NE, Yarcheski A. Alternative theories of happiness in early adolescents. Clin Nurs Res. 2002;11(3):306-23. Epub 2002/08/16. doi: 10.1177/10573802011003006. PubMed PMID: 12180642.  17. Rogers MA, Zaragoza-Lao E. Happiness and children's health: an investigation of art, entertainment, and recreation. Am J Public Health. 2003;93(2):288-9. Epub 2003/01/30. doi: 10.2105/ajph.93.2.288. PubMed PMID: 12554587; PubMed Central PMCID: PMCPMC1447731.  18. Veenhoven R. Healthy happiness: effects of happiness on physical health and the consequences for preventive health care. Journal of Happiness Studies. 2007;9(3):449-69. doi: 10.1007/s10902-006-9042-1.  19. Barfarazi H, Pourghaznein T, Mohajer S, Mazlom SR, Asgharinekah SM. Evaluating the Effect of Painting Therapy on Happiness in the Elderly. Evidence Based Care. 2018;8(3):17-26.  20. Askary P, Khayat A. The effectiveness of music therapy on severity of pain, perceived stress and happiness in adolescents with leukemia. . Positive Psychology. 2018;3(4):15-27.  21. Mirzaie M, Navidi Z. Survey personal and disease characteristics of children with cancer hospitalized in 17 shahrivar hospital. Journal of Guilan Faculty of Medicine. 2010;19(61):32-6.  22. Oliveira Pinto SM, Alves Caldeira Berenguer SM, Martins JC. Cancer, Health Literacy, and Happiness: Perspectives from Patients under Chemotherapy. Nurs Res Pract. 2013;2013:291767. Epub 2013/10/04. doi: 10.1155/2013/291767. PubMed PMID: 24089635; PubMed Central PMCID: PMCPMC3780657.  23. Khodabakhshi Koolaee A, Vazifehdar R, F, B., Akbari M. Impact of painting therapy on aggression and anxiety of children with cancer. Caspian J Pediatr. 2016;2(2):135-41.  24. Zareapour A, Falahi Khoshknab M, Kashaninia Z, Biglarian A. Effect of group play therapy on depression in children with cancer. Scientific Journal of Kurdistan University of Medical Sciences,. 2009;14(3):64-72. Persian.  25. Borzou R, Bayat Z, Salvati M, Homayounfar S. A comparison of Individual and Peer Educational Methods on Quality of life in patients with heart failure. Iranian Journal of Medical Education. 2014;14(9):767-76. Persian.  26. Philis-Tsimikas A, Fortmann A, Lleva-Ocana L, Walker C, Gallo LC. Peer-led diabetes education programs in high-risk Mexican Americans improve glycemic control compared with standard approaches: a Project Dulce promotora randomized trial. Diabetes Care. 2011;34(9):1926-31. Epub 2011/07/22. doi: 10.2337/dc10-2081. PubMed PMID: 21775748; PubMed Central PMCID: PMCPMC3161298.  27. Motevaselian M, Nasiriani K. Impact of Near-peer teaching on Learning Dressing Skill among Nursing Students. Iranian Journal of Medical Education. 2014;14(8):670-6. Persian.  28. Heisler M. Overview of peer support models to improve diabetes self-management and clinical outcomes. Diabetes Spectrum. 2007;20(4):214-21.  29. Khaw C, Raw L. The outcomes and acceptability of near-peer teaching among medical students in clinical skills. International Journal of Medical Education. 2016;7:188-94.  30. Priharjo R, Hoy G. Use of peer teaching to enhance student and patient education. Nursing Standard. 2011;25(20):3-40.  31. Michalos AC, editor. Encyclopedia of Quality of Life and Well-Being Research. 1nd ed. Dordrecht: Springer Netherlands; 2014. p. 6622-5.  32. Durualp E, Altay N. A Comparison of Emotional Indicators and Depressive Symptom Levels of School-Age Children With and Without Cancer. Journal of Pediatric Oncology Nursing. 2012;29(4):232-9.  33. Hockenberry M, Wilson D, Wong D. Wong's Essentials of Pediatric Nursing. 10^th^ ed. U.S: Mosby; 2016.  34. Michel G, Rebholz CE, von der Weid NX, Bergstraesser E, Kuehni CE. Psychological distress in adult survivors of childhood cancer: the Swiss Childhood Cancer Survivor study. J Clin Oncol. 2010;28(10):1740-8. Epub 2010/03/03. doi: 10.1200/JCO.2009.23.4534. PubMed PMID: 20194864.  35. Sanjari M, Jafarppour M, Safarabadi T, Hosseini F. Coping with cancer in teenagers and their parents. Iran Journal of Nursing. 2005;18(41):111-22. Persian.  36. Beebe A, Gelfand EW, Bender B. A randomized trial to test the effectiveness of art therapy for children with asthma. J Allergy Clin Immunol. 2010;126(2):263-6, 6 e1. Epub 2010/05/14. doi: 10.1016/j.jaci.2010.03.019. PubMed PMID: 20462632.  37. Lee S-L, Liu H-LA. A pilot study of art therapy for children with special educational needs in Hong Kong. The Arts in Psychotherapy. 2016;51(51):24-9. doi: 10.1016/j.aip.2016.08.005.  38. Barrera ME, Rykov MH, Doyle SL. The effects of interactive music therapy on hospitalized children with cancer: a pilot study. Psychooncology. 2002;11(5):379-88. Epub 2002/09/14. doi: 10.1002/pon.589. PubMed PMID: 12228871.  39. Gariépy N, Howe N. The therapeutic power of play: examining the play of young children with leukaemia. Child: care, health and development. 2003;29(6):523-37. |
